# One species does not replace a community: functional roles of co-occurring translocated and endemic mycophagous mammals

**DOI:** 10.1007/s00442-026-05947-w

**Published:** 2026-08-10

**Authors:** A. Naccarella, C. Truong, E. G. Ritchie, D. R. Sutherland, A. Coetsee, A. R. Rendall

**Affiliations:** 1 School of Life and Environmental Sciences, Deakin University, Burwood, VIC 3125 Australia; 2 Royal Botanic Gardens Victoria, Melbourne, VIC 3004 Australia; 3 School of Biosciences, University of Melbourne, Parkville, VIC 3010 Australia; 4 Phillip Island Nature Parks, Cowes, VIC 3922 Australia; 5 Wildlife, Conservation and Science, Zoos Victoria, Parkville, VIC 3052 Australia

**Keywords:** Functional redundancy, Truffle fungi, Bandicoot, Potoroo, Wallaby

## Abstract

Biodiversity decline alters species interaction networks and can destabilise ecosystem functions. Fungal consumption by mammals supports ectomycorrhizal (ECM) spore dispersal, contributing to fungi-plant symbioses. The ecological impacts of losing mycophagous mammals and to what extent species roles are redundant or complementary remains poorly quantified. Using fungal ITS2 metabarcoding, we explore the fungal community in scats of a translocated fungal generalist, the eastern barred bandicoot (*Perameles gunnii*), on Phillip and French Island in southeast Australia. We compare: (1) fungi in bandicoot scats and soil; (2) soil fungi, soil properties and vegetation to compare possible fungal availability across habitats; and (3) fungi within the scats of two sympatric mammals, an endemic fungal generalist (swamp wallaby, *Wallabia bicolor*, on Phillip Island) and fungal specialist (long-nosed potoroo, *Potorous tridactylus trisulcatus*, on French Island). We found diverging patterns between soil fungi and mammal diet; and between soil fungi, soil properties and vegetation across sites. On French Island, bandicoot and potoroo scats had similar ECM fungal richness, although community composition differed, with less ECM observed in soils. On Phillip Island, only one scat from the wallaby contained ECM fungi, while soils were more ECM diverse than bandicoot scat. Our findings suggest the functional roles of mycophages should not be considered interchangeable across ecosystems. Translocated bandicoots may have the capacity to perform functionally unique roles compared to resident mycophages, highlighting that translocations could provide multiple ecosystem benefit. Adequately assessing functional redundancy or complementarity requires a site-specific characterisation and comparison of the cumulative roles of co-occurring mycophagous mammals.

**Supplementary Information:**

The online version contains supplementary material available at https://doi.org/10.1007/s00442-026-05947-w.

## Introduction

Species and their interactions underpin the structure and resilience of ecosystems (Bascompte and Jordano 2007; Moracho et al. 2025) through critical ecosystem functions that include spore and seed dispersal, pollination or predation (Valiente-Banuet et al. 2015; Pugh and Field 2022). Maintaining or restoring species diversity contributes to network structure however species diversity alone does not necessarily preserve network integrity (Tylianakis et al. 2010). The stability and resilience of interaction networks is dependent on the diversity, complexity, and redundancy of interactions (Tylianakis et al. 2010; Landi et al. 2018). An integrated understanding of these interaction networks and ecological functions is needed to preserve or guide restoration of functionally resilient ecosystems, and by extension assess the capacity of an ecosystem to respond to environmental change, invasions, and/or extinctions (Marjakangas et al. 2025; Moracho et al. 2025).

The loss of species and subsequent simplification of communities can affect the capacity of ecosystems to maintain network structure and function. The degree to which species loss influences network structure and functions depends on the extent to which interactions are redundant or unique (Marjakangas et al. 2025). For example, the loss of a functionally unique species with an irreplaceable role will have a more substantial impact than the loss of a functionally redundant species performing a similar role to surviving species (Tylianakis et al. 2010; Cadotte et al. 2011). Thus, the conservation or reinstatement of those unique species may be prioritized to ensure the preservation of specialised interactions (Colwell et al. 2012). Conversely, restoration aimed at conserving and/or reinstating functionally redundant species can enhance ecosystem resilience and stability by buffering against future species decline or extinction (Biggs et al. 2020). The degree to which a species, and by extension their interactions, are unique or redundant is dynamic, depending on the presence and abundance of co-occurring species (Tylianakis et al. 2010), as well as environmental conditions and variation (Traill et al. 2010). Understanding which interactions are lost when species go locally or completely extinct, and by extension how reinstatement or introduction of species may return these lost interactions and functions, is critical to make informed management decisions and proactively restore ecosystem functions.

Translocations are used to reduce species extinction risk and have traditionally focused on single-species conservation goals. However, this approach is often inadequate to maintain ecosystem function and integrity (Kollmann et al. 2016; Harvey et al. 2017; Heinen et al. 2020). Restoration goals related to ecosystem functions are becoming more common with the increasing application of ecological network approaches (Lunt et al. 2013; Manning et al. 2015; Palmer et al. 2020). Thus, translocations may offer dual benefits for conservation and restoration of key interactions and functions (Seddon et al. 2012; IUCN/SSC 2013). However, there is often an implicit expectation that functional interaction networks will be restored once species are reinstated (Heinen et al. 2020). Irrespective of whether a translocation is an introduction or a reintroduction, unanticipated and unintended consequences can occur at recipient sites e.g., herbivory (Verdon et al. 2016) or reassembly of trophic dynamics (Coggan et al. 2016). Novel interactions resulting from changes in site conditions or exposure to a novel assemblage of species can further confound anticipated effects (Lunt et al. 2013; Seddon et al. 2015). The challenge of anticipating whether a translocated species has successfully reintroduced ecosystem functions and key species interactions can be exacerbated when we consider species translocated outside their historic range and/or where recipient sites are heavily degraded (IUCN/SSC 2013). To ensure that translocations achieve restoration goals, and minimize unintended consequences, we require a better understanding of how these interactions and functions operate across different species, ecosystem contexts, and spatio-temporal scales (Baker et al. 2019). The impact of translocations on ecosystems is, however, rarely monitored (Hale and Koprowski 2018; Palmer et al. 2020) and projects often neglect the complexity and context dependency of these ecosystem functions and interactions.

Understanding complex interaction networks requires a cross-taxa approach beyond two-way interactions and single-kingdom assessments (Luza et al. 2023). Digging, mycophagous (fungus-feeding) mammals are ecosystem engineers that play diverse functional roles (Fleming et al. 2014; Beca et al. 2022). Bioturbation through digging increases soil turnover, organic matter mixing, nutrient cycling, water infiltration, and creates microhabitats for seedlings and other species (e.g., invertebrates or reptiles) (Fleming et al. 2014). Through the action of digging, mammals also play a role in dispersing fungal spores, incidentally via soil movement, through direct consumption and subsequent dispersal via scats, indirectly through fur or whisker transmission, or via secondary dispersal through consumption of mycophagous invertebrates or reptiles (Elliott et al. 2024; Stephens et al. 2025). Animal-mediated spore dispersal is particularly important for truffle-like fungi that form underground fruitbodies and rely on animal consumption to actively disperse their spores (Claridge et al. 1994; Elliott et al. 2022b). Many of these fungi form ectomycorrhizal (ECM) symbiotic associations with plants, aiding nutrient and water uptake, while protecting roots from pathogens (Landeweert et al. 2001; Egerton-Warburton et al. 2007; Ghorbanpour et al. 2018). The combined influence of mammal-fungi-plant interactions and bioturbation are particularly important in resource limiting systems and in low nutrient soils, such as in Australia, where soils are highly weathered (Orians and Milewski 2007).

Species loss and subsequent simplification of co-occurring mycophagous communities is predicted to disrupt mammal-fungi-plant interactions (Vernes et al. 2015; Nuske et al. 2017; Nest et al. 2023). Fungal specialists such as species of *Potoroidae* (bettongs, potoroos and rat-kangaroos), consistently rely on fungi for the bulk of their diet throughout the year, consuming a high abundance and diversity, and therefore contributing disproportionally to spore dispersal (Bennett and Baxter 1989; Claridge et al. 1993a, b; Nuske et al. 2017). In contrast, fungal generalists (e.g., species of rodent [*Rattus* spp., *Mus* spp.], wallabies [*Wallabia bicolor*,* Macropus* spp., *Petrogale* spp.], pademelon [*Thylogale* spp.], bandicoot [*Perameles* spp., *Isoodon* spp., and *Echymipera* spp.]) consume fungi to supplement their diet depending on food availability (Claridge and Trappe 2005; Vernes 2010; Nuske et al. 2017; Danks et al. 2020; Elliott et al. 2022b). The role of fungal generalists as fungal spore dispersers therefore varies temporally (e.g., across seasons) and spatially (e.g., across sites) (Vernes et al. 2015; Nuske et al. 2017). Following extinctions that lead to the simplification of mycophagous communities, fungal generalists may become more critical for fungal spore dispersal in the absence of fungal specialists (Claridge and May 1994; Vernes 2007; Nuske et al. 2019).

We use the translocation of eastern barred bandicoot (*Perameles gunnii*) as a case study to examine and compare the fungal community within the scats of different mycophagous mammal species in southeast Australia. Bandicoot fungal diets were assessed across five translocation sites using ITS2 metabarcoding, with varying patterns of ECM fungal diversity detected at the individual-, population- and site- level, highlighting that the degree of mycophagy of a species is site-specific (Naccarella et al. 2026). Naccarella et al. (2026) results suggested that at two translocation sites located outside the eastern barred bandicoot’s historic range (Phillip and French Island), bandicoot scats contained a more diverse fungal community where other fungal specialists co-occurred (French Island), in comparison to where fungal generalists co-occurred (Phillip Island).

**Fig. 1:**
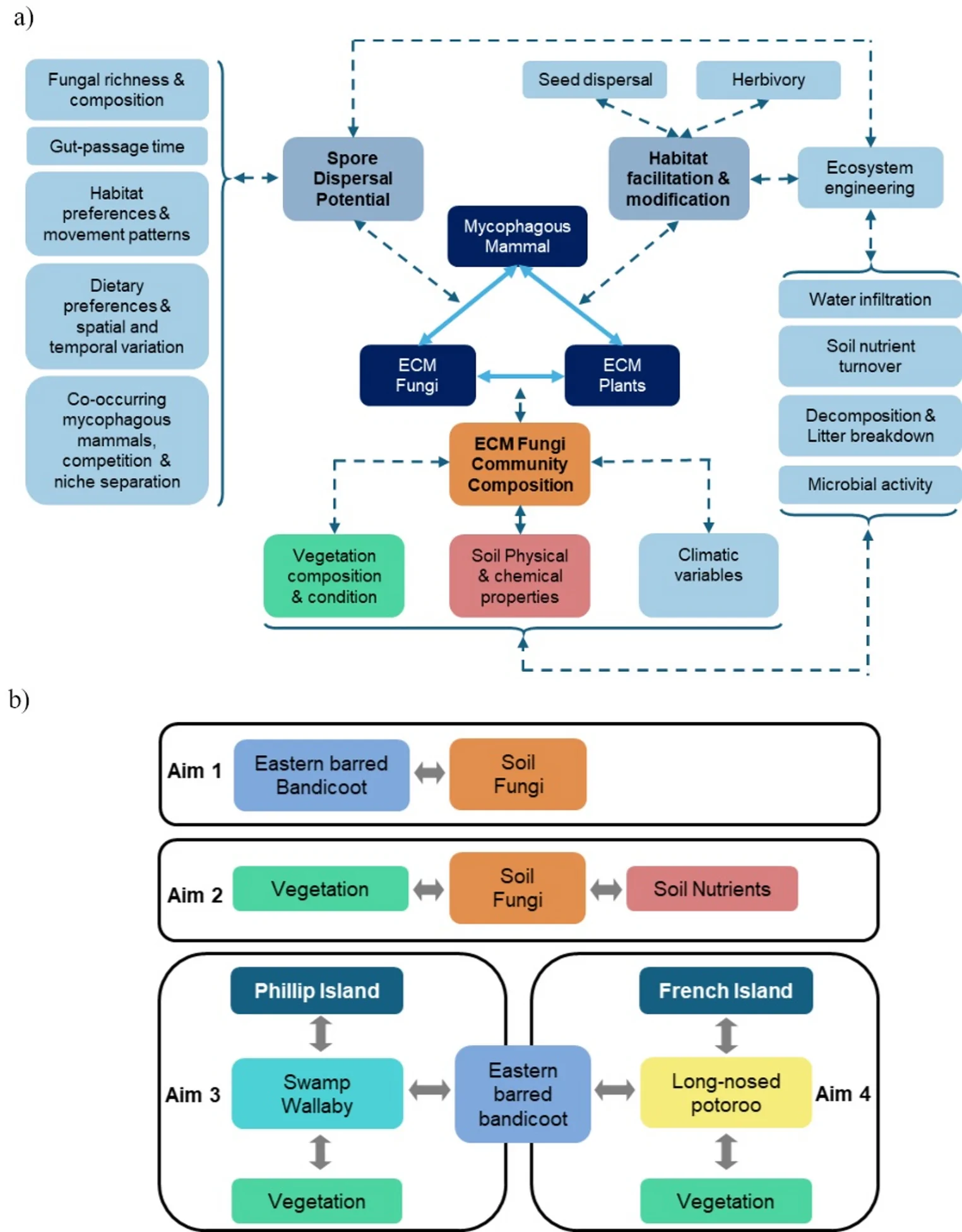
(**a**) conceptual diagram of interrelationships between mycophagous mammals, ectomycorrhizal (ECM) fungi and plant communities, and (**b**) outline of key aims explored through our research

A context-dependant understanding of mammal-fungi interactions is therefore needed to characterise fungal availability across sites and assess the uniqueness of fungal communities within eastern barred bandicoot scat and their potential contributions to spore dispersal within a wider community of mycopahgous mammals. Importantly, it is critical to understand dietary variation among co-occurring mycophagous mammals and how different species may be fulfilling different functional roles. We use these two offshore islands to explore the context-dependency of these mammal-fungal interactions and provide insights into the fungal community within the scats of the translocated eastern barred bandicoot outside their historic range. Specifically, we (Fig. 1):

1. Characterize fungal richness and community composition using ITS2 metabarcoding in soil and eastern barred bandicoot scat samples.

*We hypothesise that the diversity of ECM fungi in eastern barred bandicoot scats and in soil will be positively associated as dietary generalists are expected to consume fungi in relation to fungal availability* (Claridge and Trappe 2005; Nuske et al. 2017; Danks et al. 2020).

(2) Assess relationships between soil fungi, soil properties and vegetation to explore how site characteristics may relate to patterns in fungal communities within soil.

*We hypothesise that soil properties and vegetation will influence soil fungi communities. We anticipate that ECM fungal diversity will be positively associated with lower soil nutrients (*Lilleskov and Bruns 2001*)*,* and the abundance of ECM host plants that facilitate mycorrhizal associations.*

(3) Compare fungi in the scat of eastern barred bandicoot with an endemic fungal generalist, the swamp wallaby (*Wallabia bicolor*), on Phillip Island to assess possible differences in fungal diets and hence dispersal roles between co-occurring species. We also compare ECM fungi in scats with vegetation attributes to understand how foraging habitat may influence fungal diet patterns.

*We hypothesise that the richness and community composition of ECM in bandicoot and wallaby scats will overlap as both species are fungal generalists and thus expected to have similar fungal dietary preferences in relation to fungal availability* (Nuske et al. 2017).

(4) Compare fungi in the scats of eastern barred bandicoots with a fungal specialist, the long-nosed potoroo (*Potorous tridactylus trisulcatus*) on French Island, to assess potential fungal dispersal roles between co-occurring species. We compare the fungal community in scat with vegetation to explore how foraging may influence fungal diet patterns.

*We hypothesise that there will be differences in the richness and community composition of fungal taxonomic groups within the scats of each species*,* driven by dietary preferences and fungal specialisations. We expect that the fungal specialist*,* potoroo*,* will have a greater diversity and relative abundance of ECM and truffle-like (hypogeous) fungi within their scats*,* compared to the fungal generalist bandicoot* (Nuske et al. 2017).

## Methods

### Study sites

This study centres on two islands off the coast of Victoria in south-eastern Australia on Bunurong/Boonwurrung Country: the ‘Summerland Peninsula’ (300 ha) (38°30’42.7"S 145°08’07.9"E) site on the south-western tip of Milawul-Phillip Island (10,100 ha) (hereafter ‘Phillip Island’), and the ‘Bluegums’ (300 ha) (38°23’59.1"S 145°22’34.1"E) site in the south-east of French Island (17 000 ha) (hereafter ‘French Island’) (Figure S1.1). Eastern barred bandicoots were translocated to Phillip Island in 2017 and to French Island in 2019. Phillip and French islands experience a temperate climate (mean annual rainfall 704 mm), with warm-dry summers and cool-wet winters (Australian Bureau of Meteorology 2025). Soil type consists of a combination of vertosol and hydrosol on Phillip Island, and podosols on French Island (DEECA 2025). Both sites consist of recovering native vegetation and free of introduced red fox (*Vulpes vulpes*). Feral and domestic cats (*Felis catus*) are present, with ongoing feral cat control on Phillip Island and an eradication program underway on French Island (Rendall et al. 2022; DCCEEW 2022).

The Phillip Island site has been protected since the 1980s following reclamation from a housing estate. The site has a history of grazing over the last two centuries. Vegetation comprises *Poa* grassland and swamp paperbark (*Melaleuca ericifolia*) woodland, interspersed with stands of coastal tea tree (*Gaudium laevigatum*), drooping she-oak (*Allocasuarina verticillata*) and boobialla (*Myoporum insulare*) (Naccarella et al. 2026). Additional mycophages include the common brushtail possum (*Trichosurus vulpecula*) and black rat (*Rattus rattus*) (Vernes and McGrath 2009; Wood et al. 2015; Elliott et al. 2025; Kanishka et al. 2025).

The French Island site has been protected since 1997 when the National Park was declared. The site has a history of Chicory cropping and sheep and cattle grazing. Vegetation at the site is dominated by exotic grasses, surrounded by thickets of prickly tea-tree (*Leptospermum continentale*), and patches of sword grass (*Gahnia* spp.), austral bracken (*Pteridium esculentum*), sweet bursaria (*Bursaria spinosa*) and blackberry (*Rubus fruticosus*) (Coetsee et al. 2016; Groenewegen et al. 2017). Heathy woodlands surround the site, incorporating stands of Messmate (*Eucalyptus obliqua*), swamp gum (*E. ovata*) and manna gum (*E. viminalis*) (Naccarella et al. 2026). Additional mycophages include the bush rat (*R. fuscipies*) and swamp rat (*R. lutreolus*) (Reddell et al. 1997; Vernes and Dunn 2009). Notably, possums and larger macropods (kangaroos and wallabies) are absent from French Island (Kirkwood and Johnston 2006).

### Focal species

The eastern barred bandicoot (hereafter ‘bandicoot’) is a ~ 600–1000 g, ground-dwelling marsupial endemic to south-eastern Australia. Bandicoot are fungal generalists, with a broad diet consisting of invertebrates, fungi and plant material (Dufty 1991, 1994; Loeffler 2018; Elliott et al. 2023; Symon 2024). The mainland subspecies underwent significant population declines and range constrictions due to predation from introduced red foxes, feral cats, and habitat destruction. This culminated in the loss of the last remaining wild population in Victoria in 2002, and the species was listed as ’Extinct in the Wild’ in Victoria in 2013 (Hill et al. 2018). Reintroductions have occurred across nine sites since 1989, including introductions to three offshore islands in south-eastern Australia, situated outside the species historic range (Winnard and Coulson 2008; Hill et al. 2018; Rendall et al. 2018).

On Phillip Island, we compare the diet of bandicoot with the endemic swamp wallaby (hereafter ‘wallaby’), a 10–20 kg ground-dwelling marsupial. Wallaby are fungal generalists, feeding mainly on plant material, and supplementing their diet with fungi, largely when fungi is most abundant (Claridge et al. 2001; Vernes and McGrath 2009; Vernes 2010). Wallaby are widespread across Phillip Island, occurring at high densities in some areas (Rendall et al. 2021).

On French Island we compare the diet of bandicoot with the endemic long-nosed potoroo (hereafter ‘potoroo’), a 660–1640 g fungal specialist that consumes fungi for over 50% of their diet year-round (Claridge et al. 1993a, b; Tory et al. 1997). Potoroo have undergone significant declines, now persisting in fragmented populations across south-eastern Australia (Martin and Temple-Smith 2010). The French Island population remains a stronghold for the sub-species; however, the population undergoes fluctuations linked to feral cat densities and rainfall (Frankham et al. 2011; Eketone et al. 2026).

## Scat collection

Bandicoot and potoroo scats were collected during cage trapping for population monitoring in April 2023 using wire mesh cage traps (~ 500 × 180 × 200 mm) baited with a standard small mammal bait (see Appendix S1 for full site, trapping and sampling design). Scat was collected from the base of the trap during the first capture of an individual (as identified via a Passive Integrated Transponder tag). Multiple pellets per individual were collected where possible and mixed to form one sample per individual. Fresh wallaby scats were collected off the ground (identified based on colour, moisture and odour) by active searching within each block (see Appendix S1). Wallaby scat was collected on the same nights as trapping occurred on Phillip Island to minimise variation in fungal availability. All scats were stored within 12 h at -18 °C prior to analyses.

## Vegetation survey

We carried out vegetation surveys to evaluate the presence and abundance of ECM host plants, following methods outlined in Naccarella et al. (2026). Briefly, we measured vegetation composition (percent cover), ECM plant cover (percent cover per genera), and ECM plant basal diameter (per genera) across the trapping grids using either 20 × 20 m or 1 × 1 m quadrats (see Appendix S1 for sampling design). Plant cover and basal diameter were assessed at genus-level, enabling us to categorise plants into those that are known to form ECM associations to those that are not. Only genera known to form ECM associations were included in analyses (Soudzilovskaia et al. 2020).

## Soil sampling

Soil samples were collected at the same time as scat sampling. Soils were collected at the midpoint of each 20 m vegetation quadrat using a 5 cm wide by 10 cm deep PVC soil core (total *n* = 70) (Appendix S1, Figure S1.1). PVC soil cores were sterilised by immersion in 10% sodium hypochlorite for 10 min prior to sampling. Two soil samples were collected per quadrat and homogenised on site. Vegetation and litter directly above the soil core was cleared prior to coring. A portion of the fresh soil sample was set aside for DNA analyses and stored at -18 °C, and the remaining soil sample was oven dried at 40 °C overnight within one-week of collection for nutrient analyses. Soil nutrient analyses were undertaken by CSBP Soil and Plant Analysis Laboratory (Bibra Lake, Western Australia). Soil was analysed for ammonium nitrogen (mg/kg), nitrate nitrogen (mg/kg), phosphorus (mg/kg; Colwell), potassium (mg/kg; Colwell), sulphur (mg/kg; KCI 40), organic carbon (carbon, %; Walkley-Black), electrical conductivity (dS/m) and pH (CaCl_2_ and H_2_O).

## DNA extraction and sequencing

Scat samples were homogenised and 0.25 g per sample was used for DNA extraction. DNA extraction, PCR, sequencing protocols and bioinformatics followed methods outlined in Naccarella et al. (2026). Briefly, DNA was extracted using the Qiagen DNeasy PowerLyzer PowerSoil DNA isolation Kit, and we amplified the ITS2 region of rDNA using the forward primer gITS7ngs (GTGARTCATCRARTYTTTG) and reverse primer ITS4ngs (CCTSCSCTTANTDATATGC) (Tedersoo and Lindahl 2016). Library preparation and sequencing was conducted by EnviroDNA Pty Ltd in Melbourne, Australia (https://www.envirodna.com). DNA extractions included negative controls and PCR reactions were conducted with negative and positive controls.

### Bioinformatics

Bioinformatics were performed as per Naccarella et al. (2026). Raw ITS2 reads were processed into fungal operational taxonomic units (OTUs) at 97% sequence similarity using LotuS2 v2.34 (Özkurt et al. 2022). The taxonomy of ITS2 OTUs was assigned against UNITE v10 (McMurdie and Holmes 2013; Abarenkov et al. 2025) using blastn in LotuS2. Following initial processing in LotuS2, the ITS2 dataset was visualized in *phyloseq* (McMurdie and Holmes 2013) and putative contaminants were filtered out based on read occurrence in negative DNA extraction controls using *decontam* (Davis et al. 2018), with a cutoff threshold of 0.1, in R version 4.4 (R Core Team 2024). Following this step, OTUs with less than 0.01% of the total read count per sample were removed to account for putative index switches, and samples with less than 500 reads were discarded.

### Data analysis

All analyses were undertaken in R version 4.4.2 (R Core Team 2024), except Permutational Multivariate Analysis of Variance (PERMANOVA), which were undertaken in PRIMER version 7 (Clarke and Gorley 2015). We assigned functional guilds (ECM, arbuscular mycorrhizal (AM), endophyte, parasite/pathogen and saprotroph) based on generic identification using FungalTraits (Põlme et al. 2020). All *Glomeromycota* OTUs were designated as AM. Using FungalTraits, we also classified taxa by sporing body habit where known (hypogeous or epigeous) to assess the prevalence and diversity of truffle-like (hypogeous) taxa. We visually compared shared and unique ECM and truffle-like OTUs between sample types (soil, bandicoot, potoroo or wallaby scats) with Euler diagrams (*eulerr*, Larsson and Gustafsson 2018) and visualized fungal genera relationships via bipartite interaction matrices (*bipartite*, Dormann et al. 2008). To explore differences in ECM genera between sample types (soil, bandicoot, wallaby and potoroo scats) and sites (Phillip and French islands) we performed Indicator-Species Analysis using *indicspecies* with 99,999 permutations (Cáceres and Legendre 2009). We report the overall Indicator Value (IV), in addition to Component A (specificity; mean abundance of genera in the target sample types divided by the sum of the mean abundance values over all sample types) and Component B (fidelity; the relative frequency of occurrence of the genera in the target sample type) separately to explore differences between fungal genera that are unique compared to those that are dominant.

For each aim, we used Generalised Linear Models (GLM), Linear Mixed Models (LMM), Generalised Linear Mixed Models (GLMM) or Generalised Additive Models (GAM) to assess ECM richness metrics. We assessed model fit using simulated residual diagnostics (*DHARMa* package; Hartig et al. 2024) and evaluated outliers via cook’s distance (see Appendix S2). Significant interactions were explored using Tukey’s tests with adjusted p-values for multiple comparisons in *emmeans* (Lenth 2016). For Aims 1 and 4 we ran separate models for three richness metrics; ECM and truffle-like observed fungal richness (number of ECM fungal OTUs per sample) and, ECM Shannon diversity. For models which assessed soil properties or vegetation variables ECM richness was used as the response variable.

For each aim we ran PERMANOVAs or distance-based Redundancy Analyses (db-RDAs) to assess ECM fungal community composition using Raup-Crick dissimilarity matrices on presence/absence data (Raup and Crick 1979; Lenth 2016) that are well-suited to compare communities with varied species and sample sizes (Chase et al. 2011; Staudhammer et al. 2018). We performed pairwise comparisons when interactions were significant. We additionally used PERMDISP to test for homogeneity of group dispersions and where significant ran all pairwise combinations to explore differences. For models including environmental variables (soil or vegetation via db-RDA) we modelled explanatory variables using Euclidian dissimilarity matrices. We used *bioenv* (*vegan*, Oksanen et al. 2020) to identify the best possible rank-order correlation with the ECM fungal community. When *bioenv* correlations were > 0.2 we assessed significance with Analysis of Variance (ANOVAs) and ran db-RDAs.

Prior to analyses, environmental variables that correlated strongly with one another (Pearson correlation > 0.70 or < -0.70) were identified and one of each pair was removed. We retained soil properties known to have a stronger influence on soil fungal communities as well as vegetation variables with greater percent cover and/or detections (see Supplementary Material). For all models including vegetation attributes we ran models for each scale separately, including: vegetation composition (percent cover), ECM plant cover (percent cover per genera), and ECM plant basal diameter (per genera). Only plant genera that were present in two or more quadrats were included in analyses. For all models, environmental variables were scaled and centred, and we ran all possible combinations of predictors and ranked models using Akaike’s Information Criterion (AICc), corrected for small sample size, considering models with ΔAICc < 2 to have support.

Models for each aim were as follows (see Appendix S2 for details):

- *Aim 1*: We compared patterns in ECM richness and community composition between sample types (bandicoot and soil) and sites (Phillip and French islands), with site, sample type and their interaction included as fixed effects, and block as a random effect.
- *Aim 2*: To understand how ECM fungi in soil relate to environmental variables we ran models for soil properties and each of the vegetation metrics (composition, cover and basal diameter). All metrics were assessed at the 20 × 20 m quadrat scale (rather than block) as soil samples were collected per quadrat. For richness models we used ECM richness as the response variable, site was included as a fixed effect and as an interaction term with each explanatory variable. For plant cover and ECM plant basal diameter we ran models for each island separately due to variations in the dominant genera at each site (Appendix S5). For the community composition models db-RDAs were run per environmental variable and separately for each island based on differences in the fungal communities between sites identified via Aim 1.
- *Aim 3*: Due to minimal detections of ECM fungi in wallaby scats we did not compare bandicoot and wallaby fungal diets. To explore relationships between vegetation attributes and the bandicoots’ fungal diet on Phillip Island, we ran separate models across the three vegetation scales (composition, cover and basal diameter). In each model block was included as a random effect. For the ECM richness models, due to high correlation between vegetation composition attributes, we used Principal Components Analysis (PCA) to summarise trends and included components that cumulatively explained 80% or more of the variation. For the ECM plant cover and ECM plant basal diameter models we ran a model for each vegetation attribute separately to avoid overparameterization due to low bandicoot sample sizes. For the community composition models, we used partial db-RDAs controlling for the effect of block.
- *Aim 4*: To compare bandicoot and potoroo fungal diets in relation to soil on French Island, we ran three separate richness models for; ECM observed fungal richness, ECM Shannon diversity index and truffle-like observed fungal richness, and a community composition model via PERMANOVA. In all models, sample type (bandicoot, potoroo or soil) was included as fixed effect and block as a random effect. To understand relationships between vegetation attributes and the bandicoots and potoroos fungal diet we followed methods in Aim 3. We ran separate models across the three vegetation scales (composition, cover and basal diameter) against ECM richness. For the community composition models, we used partial db-RDAs controlling for the effect of block.

## Sample size

To account for the uneven sample sizes between sites and sample types, we used the pseudo-multivariate dissimilarity based standard error (MultSE; Anderson and Santana-Garcon 2015) to assess whether sample size was adequate to capture observed differences. For each site we calculated the MultSE estimates and bootstrapped error estimates for soil and scat samples using all OTUs, and ECM OTUs specifically (Appendix S3). As diversity indices can be biased by both sample size and sampling depth, we support these interpretations by assessing the accumulation of OTUs per site and sample type across sequence read counts (depth) using rarefaction OTU accumulation curves for ECM and truffle-like taxa (‘*ggrare’*, richness.R, phyloseq extensions; Mariadassou 2019).

## Results

**Fig. 2:**
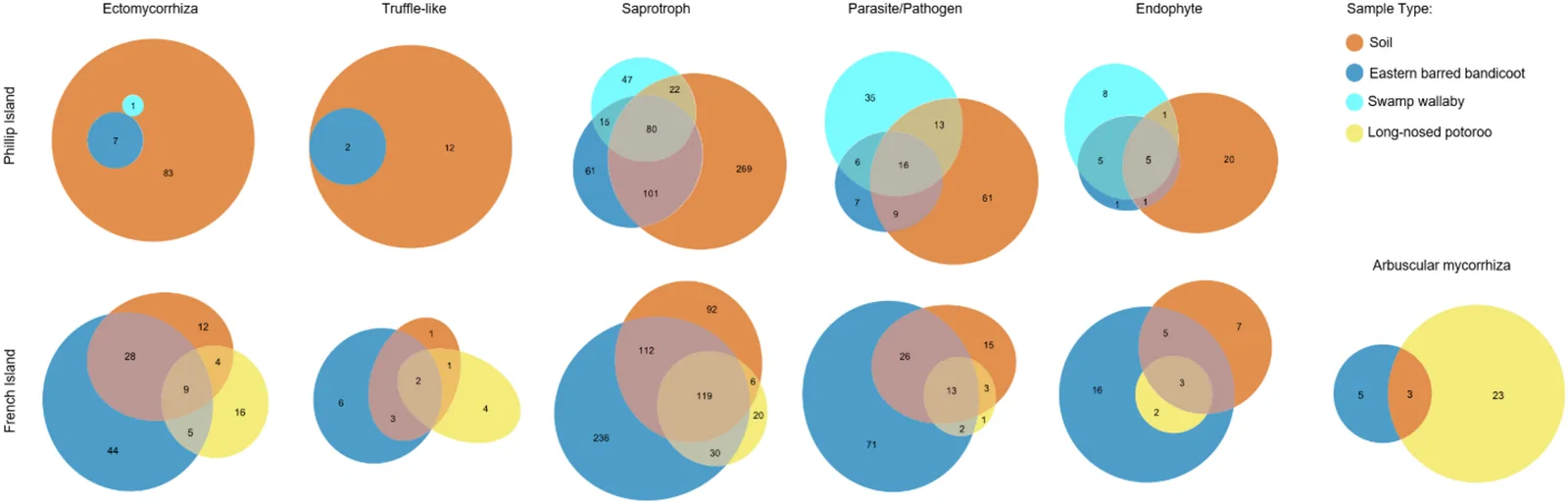
Euler diagrams of shared and unique taxa from each functional guild in soil, eastern barred bandicoot (*Perameles gunnii*) scat, long nosed potoroo (*Potorous tridactylus trisulcatus*) scat and swamp wallaby (*Wallabia bicolor*) scat collected from Phillip (a-e) and French Islands (f-k). The size of a circle represents the relative richness of each sample. Overlapping areas represent the proportion of shared operational taxonomic units between samples, and the non-overlapping areas represent unique OTUs. Numbers inside the circles represent number of shared taxa. Total sequenced sample size: Phillip Island: bandicoot scat *n* = 8, wallaby *n* = 20, soil *n* = 29. French Island: bandicoot scat *n* = 37, potoroo scat *n* = 6, soil *n* = 38 soil. Arbuscular mycorrhiza were only detected in soil samples from Phillip Island and thus were not plotted

We collected 11 bandicoot and 20 wallaby scats from Phillip Island and 40 bandicoot and 6 potoroo scats from French Island (Appendix S3). A total of 40 soil samples were collected from French Island and 30 from Phillip Island. After filtering samples with < 500 reads, 138 samples were retained for further analyses (French Island: 37 bandicoot, 6 potoroo, 38 soil; Phillip Island: 8 bandicoot, 20 wallaby, 29 soil). 3,811 filtered fungal operational taxonomic units (OTUs) were identified (mean 213.5 ± 121.8 SD OTUs, range 19–585). Overall, 81% of OTUs were assigned to class, 71% to order, 60% to family, 49% to genus and 29% to species. At generic levels using FungalTraits, the greatest number of OTUs were saprotroph (790 OTUs across 138 samples), followed by parasite/pathogen (172 OTUs across 131 samples), ECM (136 OTUs across 83 samples), AM (Glomeromycota; 46 OTUs across 42 samples), endophyte (50 OTUs across 119 samples), and truffle-like ECM (20 OTUs across 48 samples; Fig. 2). In total we detected 29 ECM genera, with the highest richness in *Sebacina* (17 OTUs), *Tomentella* (16 OTUs), *Russula* (12 OTUs) and *Inocybe* (11 OTUs). Eleven truffle-like ECM genera were identified, with over half of truffle-like OTUs assigned to *Ruhlandiella*, and one truffle-like taxa was identified to species level, *Hydnoplicata convoluta*. The MultiSE and rarefaction OTU accumulation curves indicated that samples did not reach an asymptote, suggesting that additional samples are required to fully capture dietary breadth and fungal availability across both sites (Appendix S3).

### Aim 1: Bandicoot fungal diet in relation to soil fungal availability

**Fig. 3:**
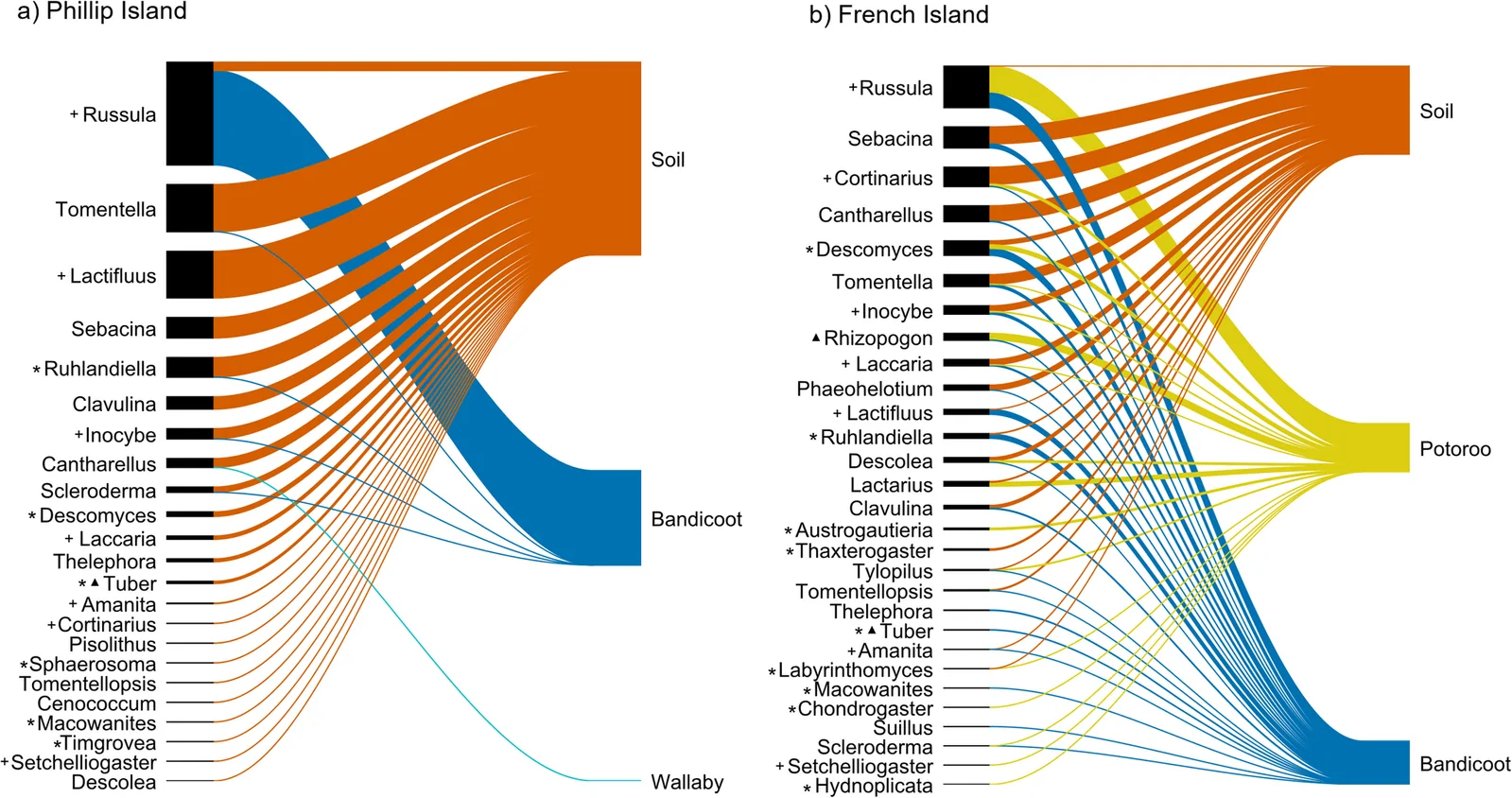
Bipartite plot of the ectomycorrhizal genera in soil, eastern barred bandicoot (*Perameles gunnii*) scat, long-nosed potoroo (*Potorous tridactylus trisulcatus*) scat and swamp wallaby (*Wallabia bicolor*) scat collected from Phillip and French Island, Victoria, southeastern Australia. The size indicates the relative abundance per genera. Total sequenced sample size: Phillip Island: bandicoot scat *n* = 8, wallaby *n* = 20, soil *n* = 29. French Island: bandicoot scat *n* = 37, potoroo scat *n* = 6, soil *n* = 38 soil. * = genera with species that exclusively have a truffle-like (hypogeous) fruiting habit. + = genera with species being either epigeous (mushroom-forming) or with a truffle-like fruiting habit. ▲ = genera that are exotic to Australia. Taxonomy follows UNITE v10 (Abarenkov et al. 2025)

We found different trends in ECM fungal richness, Shannon diversity and truffle-like richness between soil and bandicoot samples, and these differed across sites (Fig. 3, Appendix S4). On Phillip Island, soil samples had a higher ECM richness and Shannon diversity than bandicoot scats (Table 1). Conversely on French Island, bandicoot scats had a higher ECM richness and Shannon diversity than soil (Table 1). There was no difference in truffle-like richness between bandicoot and soil samples across sites. Soil on Phillip Island had higher ECM and truffle-like richness than on French Island (Table 1). There was no difference in Shannon diversity between sites. Conversely, we found a higher ECM richness and Shannon diversity in bandicoot scats from French Island compared to Phillip Island (Table 1), yet no difference in truffle-like richness between sites.

**Fig. 4:**
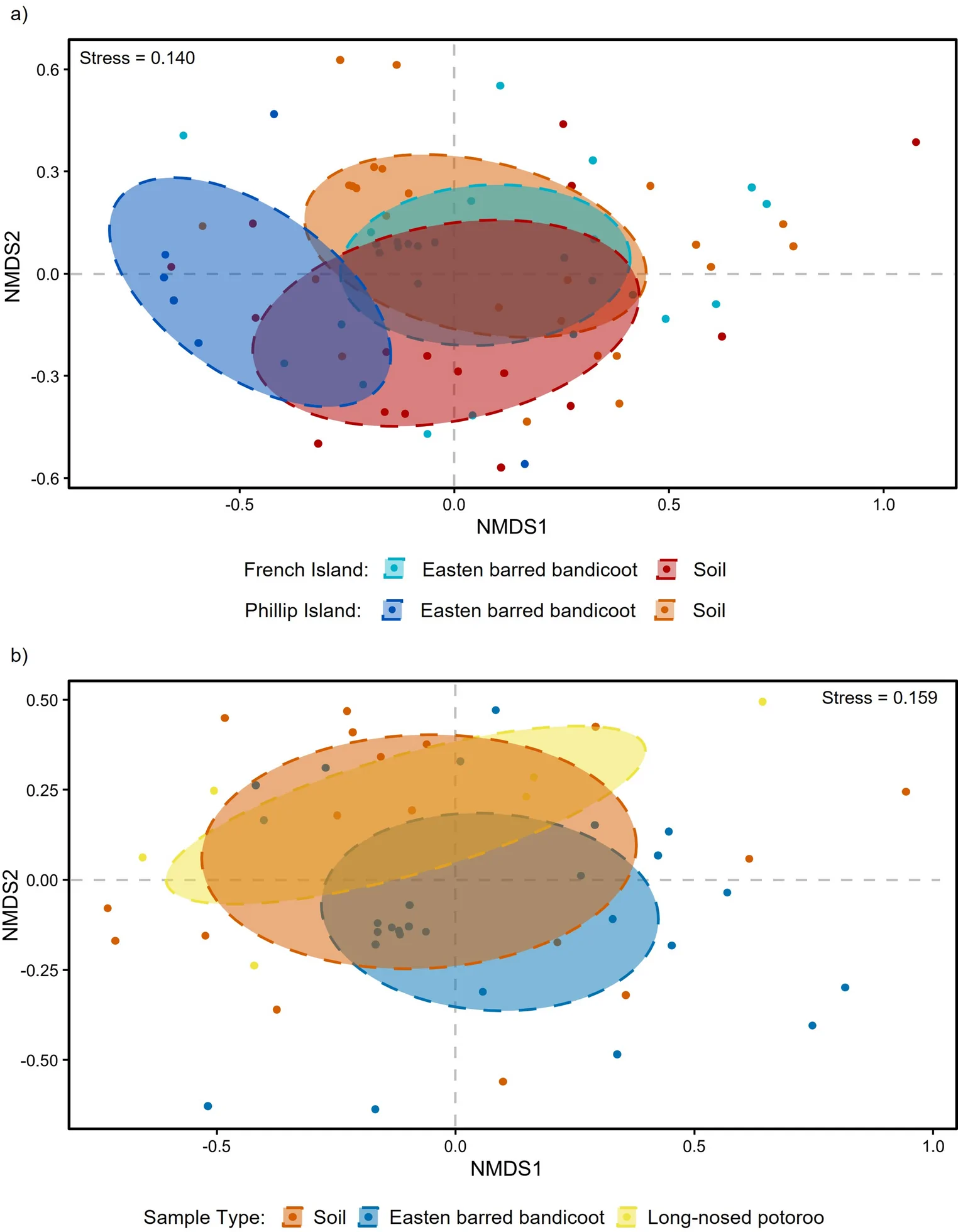
Non-metric multidimensional scaling (NMDS) ordination of ectomycorrhizal fungal communities (using Raup-Crick dissimilarities based on presence/absence) in (**a**) soil and eastern barred bandicoot (*Perameles gunnii*) scat collected from French and Phillip Island and (**b**) soil, eastern barred bandicoot and long-nosed potoroo (*Potorous tridactylus trisulcatus*) scat collected from French Island. Dashed lines represent 95% confidence interval

We detected differences in the ECM fungal community between soil and bandicoot samples, and between Phillip and French Islands (Fig. 4, Appendix S4). We recorded differences between soil samples from Phillip and French Island in the ECM (F = 0.076, df = 1, *p* = 0.022) and truffle-like community (F = 6.596, df = 1, *p* = 0.005) however no difference in community breadth (Appendix S4). There was no difference between ECM community composition in bandicoot scats between sites (Appendix S4). We detected differences in community composition (t = 1.765, *p* = 0.033) and no difference in breadth between soil and bandicoot samples on Phillip Island (Appendix S4). On French Island we found no difference in ECM nor truffle-like community composition or breath between bandicoot and soil samples (Appendix S4).

Indicator species analysis identified nine genera characterising five site group combinations (Appendix S4). *Russula* was the strongest indicator of bandicoot scats from Phillip Island (IV = 0.885), and *Tomentella* of bandicoot scats from French Island and soil samples from both sites (IV = 0.828). All other site-group combinations were strongly driven by Component A, suggesting that these genera were exclusive to these sites with low occurrences. One truffle-like genus, *Ruhlandiella* , was indicative of bandicoot samples from both sites and soil samples from Phillip Island (IV = 0.827).

### Aim 2: Relationships between soil fungi and environmental variables

#### Soil properties

The best supported model explaining the relationship between ECM fungal richness and soil properties included site, ammonium nitrate, nitrate nitrogen, organic carbon, the interaction of site with nitrate nitrogen, and the interaction of site with ammonium nitrate (Appendix S5). High observed ECM richness within soil samples was associated with lower ammonium nitrate across both sites, and lower nitrate nitrogen on Phillip Island, although there was uncertainty at lower values (Appendix S5).

We found associations between the ECM fungal community assemblage and soil properties at both sites (Fig. 4). Ammonium nitrate, nitrate nitrogen, phosphorous, and potassium were the best correlates of ECM community in soil samples from Phillip Island (*bioenv* = 0.225; Appendix S5), explaining 26.99% of variation in the community. Phosphorous was the most influential driver (F = 3.103, *p* = 0.011). On French Island, phosphorous, organic carbon and pH were the best correlates of the ECM community in soil samples (*bioenv* = 0.375), explaining 29.94% of variation in the community. There were no significant drivers, with pH showing a marginal trend (F = 2.402, *p* = 0.067; Appendix S5).

### Vegetation attributes

The best supported model explaining the relationship between ECM richness within soil and vegetation composition included bare ground, woody vegetation, herb and litter cover (Appendix S5). Litter was deemed an uninformative parameter as per Leroux (2019). Higher ECM richness was associated with higher percent cover of woody vegetation and higher percent cover of bare ground across both sites, however there was variability on Phillip Island (Table 1, Appendix S5). High percent cover of herb was associated with lower ECM richness in soil from Phillip Island however this was largely driven by a single sample (Table 1, Appendix S5).

**Table 1:** Summary of influential variables and comparisons of sample types, across all four aims. All non-influential results are reported in supplementary material. Aim 1: Bandicoot fungal diet in relation to soil availability; Aim 2: Relationship between soil fungi and environmental variables; Aim 3: Comparison of fungal generalists on Phillip Island

Aim	Response	Island	Key Results (β, SE, *p*)
1	ECM Richness	Phillip	Soil > Bandicoot ( -2.400, 0.710, < 0.001)
		French	Bandicoot > Soil (1.350, 0.386, < 0.001)
	ECM Shannon Diversity	Phillip	Soil > Bandicoot (-1.201, 0.436, 0.008)
		French	Bandicoot > Soil (0.741, 0.307, 0.018)
	Soil ECM Richness		Phillip > French (-1.850, 0.391, < 0.001)
	Soil Truffle-like Richness		Phillip > French ( -1.120, 0.320, < 0.001)
	Bandicoot ECM Richness		French > Phillip (1.910, 0.692, 0.006)
	Bandicoot ECM Shannon Diversity		French > Phillip (1.375, 0.442, 0.003)
2	Soil ECM richness - Soil Properties	Phillip and French	Higher ECM with lower ammonium nitrate (-0.834, 0.207, < 0.001)
		Phillip	Higher ECM with lower nitrate nitrogen (2.017, 0.826, 0.015)
	Soil ECM richness – Vegetation Composition	Phillip and French	Higher ECM with higher woody vegetation (0.018, 0.007, 0.012) Higher ECM with higher bare ground (0.110, 0.036, 0.002)
		Phillip	Higher ECM with lower herb (-0.278, 0.100, 0.005)
	Soil ECM richness – ECM Plant Cover	French	Higher ECM with higher *Eucalyptus* (0.165, 0.043, < 0.001) Higher ECM with higher *Melaleuca* (0.056, 0.009, < 0.001)
	Soil ECM richness – ECM Plant Basal diameter	French	Higher ECM with greater *Eucalyptus* (0.671, 0.195, 0.001) Higher ECM with greater *Melaleuca* (0.985, 0.213, < 0.001)
3	Bandicoot ECM richness - Vegetation Composition	Phillip	Higher ECM with lower graminoid, higher woody debris and herb (-0.344, 0.164, 0.036)
	Bandicoot ECM richness - ECM Plant Cover	Phillip	Higher ECM with higher *Acacia* (0.225, 0.105, 0.032) Higher ECM with higher *Gaudium* (0.248, 0.122, 0.042)

On Phillip Island ECM richness within soil was influence by *Melaleuca* cover (AICc ω = 0.187; Appendix S5), however the null model also received support (ΔAICc = 0.500). Higher *Melaleuca* cover trended towards higher ECM richness (β = 0.445; 95%: -0.044 to 1.031). ECM richness within soil was influenced by *Melaleuca* and *Allocasuarina* basal diameter (Appendix S5). Basal diameter of *Melaleuca* suggested an increase in ECM richness until moderate values before declining again (Figure S5.4). There were no associations between ECM community assemblage and vegetation on Phillip Island, with all *bioenvs* < 0.2 (Table S5.18).

On French Island soil ECM richness was influenced by *Eucalyptus*, *Melaleuca*, *Leptospermum* and *Gaudium* cover (Appendix S5). Higher *Eucalyptus* and *Melaleuca* cover were associated with higher ECM richness (Table 1, Table S5.21). Soil ECM richness was influenced by the basal diameter of *Eucalyptus*, *Leptospermum* and *Melaleuca* (Appendix S5). Greater basal diameter of *Eucalyptus* and *Melaleuca* was associated with higher ECM richness (Table 1). Greater basal diameter of *Leptospermum* trended towards higher ECM richness (β = 0.371; 95%: 0.019 to 0.850). Herb, woody vegetation, canopy and woody debris cover were the best correlates of the ECM community in soil (*bioenv* = 0.268; Table S5.25), explaining 26.39% of variation in the community. However, none of these were significant drivers (Appendix S5). *Melaleuca*, *Leptospermum*, and *Eucalyptus* cover were the combination of ECM plants most correlated with the ECM community in soil samples (*bioenv* = 0.213; Table S5.28), explaining 30.99% of variation in the community. Of these the percent cover of *Melaleuca* was the most influential driver (F = 3.745, *p* = 0.012). There were negligible trends for ECM plant basal diameter with the most parsimonious model including *Melaleuca*, *Eucalyptus* and *Gaudium* (*bioenv* = 0.116; Table S5.31).

### Aim 3: Comparison of fungal generalists on Phillip Island

We recorded a single ECM OTU (*Cantharellus*) from a single wallaby sample (Fig. 3). The number of reads and relative abundance of this OTU was negligible in comparison to overall ECM OTUs recorded in bandicoot and soil samples from Phillip Island. We detected endophyte, parasite/pathogen and saprotroph in several wallaby samples. Endophyte and saprotroph relative abundance were comparable with bandicoots from Phillip Island, while wallaby samples had the highest relative abundance of parasite/pathogen. Bandicoot samples had higher relative abundance of ECM fungi than soil samples, however the opposite was found for truffle-like fungi. Both ECM and truffle-like fungi were detected in a higher proportion of soil (ECM: 90%, truffle-like: 52%) compared to bandicoot scat (ECM: 86%, truffle-like: 14%) (Fig. 2, S4), with only one bandicoot scat containing truffle-like fungi (Figure S3.5). The number of shared ECM and truffle-like OTUs between scat (bandicoot and wallaby) and soil was high, with all OTUs detected in scat also recorded in soil. For saprotroph, parasite/pathogen and endophyte OTUs we found similar patterns to ECM in that soil samples had the greatest number of unique OTUs, however across all guilds there was both overlap and unique OTUs in bandicoot and wallaby samples (Fig. 3, Appendix S6). Arbuscular mycorrhiza was only detected in soil from Phillip Island.

Comparing ECM fungi within bandicoot scats and vegetation composition we identified two distinct vegetation gradients, explaining 87.5% of the total variation among samples (Table S6.1). PC1 (58%) positively correlated with bare ground, litter, woody vegetation and canopy cover, and PC2 (29%) positively correlated with graminoid and negatively correlated with woody debris and herb cover. PC2 showed a negative association with ECM richness, with lower graminoid cover and higher woody debris and herb cover associated with higher ECM richness (Table 1). ECM fungi in bandicoot scats were influenced by *Acacia* and *Gaudium* cover, and were associated with higher ECM richness (Table 1). There were no trends between ECM plant basal diameter and ECM richness (Table S6.4). There were strong associations between ECM community assemblage and vegetation on Phillip Island. The most parsimonious models included woody debris (*bioenv* = 0.451), and woody vegetation and woody debris (*bioenv* = 0.451; Table S6.5). For the ECM plant model including *Gaudium* and *Melaleuca* cover, the most parsimonious model included *Gaudium* (*bioenv* = 0.501), and for the model including *Acacia* and *Melaleuca*, the most parsimonious model included *Melaleuca* (*bioenv* = 0.415; Table S6.6). We were unable to run ANOVAs or db-RDAs due to only having a single factor. 

### Aim 4: Comparison of a fungal generalist and specialist on French Island

On French Island, ECM and truffle-like OTUs were detected in a higher proportion of scat (ECM: 73% of bandicoot and 100% potoroo scats, truffle-like: 49% of bandicoot and 67% of potoroo) than soil (ECM: 45%, truffle-like: 26%; Fig. 3). We found similar patterns on French to Phillip Island for saprotroph and parasite/pathogen OTUs with some overlap across sample types (Fig. 2). For endophyte we found no unique OTUs in potoroo samples and for arbuscular mycorrhiza no unique OTUs in soil samples. In contrast to Phillip Island, we found the greatest number of saprotroph, parasite/pathogen and endophytes OTUs in bandicoot scat, and for arbuscular mycorrhiza the greatest number of OTUs in potoroo scat.

We found no differences in observed ECM richness, Shannon diversity or observed truffle-like richness between bandicoot and potoroo scat, nor between potoroo and soil (Appendix S7). We detected differences in ECM (Pseudo-F = 4.094, df = 2, *p* = 0.003) and truffle-like (Pseudo-F = 3.484, df = 2, *p* = 0.031) community composition between sample types (Fig. 4). ECM (t = 2.500, *p* = 0.003) and truffle-like (t = 2.690, *p* = 0.019) community composition differed between bandicoot and potoroo scat (Table S7.4), and there were trends towards differences between potoroo and soil (t = 1.779, *p* = 0.052). No differences in community breadth were recorded, however there were trends towards differences between soil and potoroo scat (t = 1.779, *p* = 0.052; Table S7.4).

The best supported models explaining the relationship between ECM richness and vegetation composition, ECM plant cover and ECM plant basal diameter, in relation to sample type (bandicoot or potoroo), were the null models indicating that vegetation was not a good predictor of richness (Appendix S7). We found no strong correlations in community composition and vegetation variables, with *bioenv* correlations < 0.2 across all models (Table S7.11). Indicator species analysis identified a single genus characterising bandicoot samples (*Lactifluus*, IV = 0.636) and five genera characterising potoroo samples. Of these, *Russula* (IV = 0.862) and *Lactarius* (IV = 0.790) were the strongest indicators (Table S7.12). The remaining three genera (*Rhizopogon*, *Labyrinthomyces*, *Tylopilus*) were more strongly driven by Component A, suggesting that these genera were exclusive to potoroo yet had lower occurrences. *Labyrinthomyces* was the only truffle-like genera identified as an indicator of potoroo samples.

## Discussion

The decline and extinction of mammal species and subsequent simplification of mycophagous communities can disrupt mammal-fungi-plant interaction networks and ecosystem functions (Vernes 2010; Vernes et al. 2015; Danks et al. 2020; Nest et al. 2023). Translocations of mycophagous mammals have the capacity to reinstate lost ecosystem functions, albeit contingent on how unique or redundant the species are with respect to fungal spore dispersal and how this might vary across different ecosystems. By simultaneously comparing the ECM fungi in the scats of translocated bandicoots with soil fungal communities, and with the scats of co-occurring endemic mycophagous mammals (wallaby and potoroo), our current study highlights that bandicoots may have the capacity to perform unique ECM spore dispersal roles. On Phillip Island we detected low fungal diversity in bandicoot scats, although more substantial when compared to the single ECM taxon detected in a single wallaby scat. On French Island, bandicoot scat had a different composition of ECM fungi when compared to potoroo scat, however a comparable level of diversity. We acknowledge that additional sampling is required to fully capture fungal breadth, however insufficient sample size can be common for studies assessing fungal communities via DNA metabarcoding, particularly in relation to scat samples (Nuske et al. 2018). Additionally further assessments are required to explore dietary breadth (i.e., plant material and invertebrates) and if fungal detections correlate with successful fungal spore dispersal (i.e., microscopy and spore viability). Nevertheless, the high level of variability at individual-, population- and site- scales suggests that the degree of mycophagy is not consistent across a species or populations of the same species. Hence community-level assessments are required to understand the realised ecological contribution of translocated species to ecosystem function (Vernes et al. 2015; Nuske et al. 2017; Nest et al. 2023).

### Aim 1: Bandicoot fungal diet in relation to soil ECM fungal availability

Contrary to our initial hypothesis, we found diverging trends between sites in the richness of ECM and truffle-like fungi in soil compared to bandicoot scat. We recorded higher ECM richness in soils from Phillip Island compared to French Island. Based on Naccarella et al. (2026) we expected soil from Phillip Island to be depauperate of ECM and truffle-like taxa as we recorded a low diversity within bandicoot scats, and generalists are thought to opportunistically consume fungi in proportion to their relative abundance (Vernes et al. 2015; Nuske et al. 2017). Two of the prominent ECM indicators of soil and/or scat across both sites, *Ruhlandiella* and *Tomentella*, are genera known to occur in disturbed habitats (Warcup 1991; Jairus et al. 2011; Sugiyama and Sato 2021). Bandicoot diet may therefore reflect the disturbed nature of the site and these genera may not form the dominant component of the bandicoot’s fungal diet in other ecosystems. The diverse ECM and truffle-like taxa recorded in soil samples suggests that overall fungal resources are not limiting, this may suggest that the fungi observed in scats may be more reflective of sporing body availability, seasonality, or alternative food resources at the time of sampling on Phillip Island (*sensu* Naccarella et al. 2026).

We found lower ECM richness in soil compared to bandicoot scat and different fungal communities in bandicoot scat and soil samples from French Island. The lower ECM diversity in soil samples is surprising given that soils tend to consistently show higher ECM richness than scat (Nuske et al. 2019; Gil-Fernández et al. 2025). This may suggest that bandicoots on French Island were not consuming fungi in direct relation to their availability, that our soil sampling did not fully characterise fungal availability, and/or that bandicoots foraged outside the sampling area. However, given that 70% of ECM taxa detected in soil were detected in bandicoot scat, yet half of the ECM taxa recorded in bandicoot scats were unique, this may indicate a level of selectivity or habitat selection resulting in bandicoot preferencing specific taxa. Similar trends have been recorded in the fungal generalist swamp wallaby which showed preferences for rare or undetected taxa in sporocarp surveys (Danks 2011). Given the variability across fungal generalists, relating sporocarp availability to diet may provide a more comprehensive understanding of dietary preferences than soil samples.

The use of soil samples as a metric of fungal availability, concurrently with dietary assessments, poses several challenges. For example, species presence may reflect mycelium or relict spores (Carini et al. 2016; Okada and Matsuda 2022), and it is not possible to assess whether sporing bodies were available for consumption at the time of sampling. Mammal scats have been suggested as a proxy for assessing current fungal availability, the application of which warrants further investigation, particularly as a pre-assessment tool for translocation of mycophages for fungal and mammal conservation (Bradshaw et al. 2022; Gil-Fernández et al. 2025; Quah et al. 2025). Furthermore, while spores have been found to remain viable after passage through the mammalian gut (Claridge et al. 1992; Reddell et al. 1997; Tay et al. 2018), we did not assess spore viability via microscopy or inoculation trials. Our results therefore indirectly infer the potential for the dispersal of fungal tissue and/or spores within scats. Further studies combining multiple sampling methods (DNA metabarcoding, microscopy, spore viability assessments and sporocarp surveys) would provide greater insights into these mammal-fungi interactions.

### Aim 2: Relationships between soil fungi and environmental variables

Higher ECM richness in soils was associated with lower ammonium nitrate across both sites, and lower nitrate nitrogen on Phillip Island, broadly aligning with our hypothesis. Elevated nitrogen has been linked to reductions in ECM richness and abundance (Wallander 1995; Lilleskov and Bruns 2001; Janssens et al. 2010). Critically for mammal diets, several studies have found negative influences of nitrogen on sporocarp production and mycorrhizal root colonization (Lilleskov and Bruns 2001; Avis et al. 2003; Janssens et al. 2010; Bashian-Victoroff et al. 2025), impacting sporing body production before declines in soil-fungal richness are observed (Peter et al. 2001). The high uncertainty at lower levels of ammonium nitrate and nitrate nitrogen that we observed suggests that the impacts of elevated nutrients may be more influential at higher concentrations. Most previous studies, however were conducted in temperate regions of the northern hemisphere, and the impacts of elevated nutrients via anthropogenic fertilisation may be more pronounced in Australia’s nutrient poor soils (Orians and Milewski 2007).

We found distinct patterns between soil nutrients and soil ECM community across sites (Lilleskov and Bruns 2001; Danks et al. 2013). Different ECM fungal species are adapted to different nutrient conditions (Lilleskov et al. 2002; Hobbie and Agerer 2010). For example, in southeastern Australia, different truffle-like taxa assemblages were found in areas of high soil nitrogen or phosphorous (Johnson 1994; Claridge et al. 2000). Phosphorous was the most significant predictor of ECM community composition on Phillip Island and is known to be associated with lower ECM fungal richness and to impact the formation of ECM mycelia and sporocarps (Bougher et al. 1990; Wallander and Nylund 1992; Wallander 1995). In contrast on French Island, we found community associations with phosphorous, organic carbon and pH, yet none showed strong trends. Since elevated levels of soil nutrients select for different fungal species, there may be both individual and synergistic effects of nitrogen and phosphorous (Bashian-Victoroff et al. 2025). Both sites have experienced disturbance through historic land uses (e.g., housing estates and agriculture) although remain long-unburnt. Disturbances can impact ECM (Newbound et al. 2012) through soil compaction and changes to soil physiochemical properties (Hartmann et al. 2014). Whether it is the mechanism of high or low soil nutrients, or legacy effects from historic land-use which is driving these patterns remains unknown and requires further investigation (Wallander 1995; Trudell and Edmonds 2004).

Woody vegetation was a strong predictor of soil ECM richness across both sites in line with our hypothesis. Higher cover and basal diameter associated with increased richness, as ECM plant diversity and density facilitates the diversity and relative abundance of ECM fungi (Tedersoo et al. 2010). We found positive associations between bare ground and ECM richness, and a negative association between herb and soil ECM. This may reflect limited understory growth in woodland areas and/or a dominance of AM fungi which associate with herbs and grasses. Basal diameter of *Eucalyptus* (French Island) and *Melaleuca* (French and Philip Island) was associated with higher ECM richness despite exhibiting high variation between samples. *Melaleuca* and *Eucalyptus* are dual AM-ECM mycorrhizal plants (Teste et al. 2020; Bermúdez-Contreras et al. 2020). *Eucalyptus* is generally expected to transition from AM to ECM dominance as the plant ages (Chen et al. 2000; Churchland and Grayston 2014), while AM-ECM associations in *Melaleuca* can shift temporally and spatially based on conditions (Florence et al. 2025). Fungal associations in both genera can be influenced by environmental conditions, such as poor drainage (Tedersoo and Bahram 2019; Teste et al. 2020; Florence et al. 2024) or dry conditions (e.g., Adjoud-Sadadou and Halli-Hargas 2017), which may shift ECM dominance and diversity. We note that higher woody vegetation cover coincided with lower nitrate nitrogen and ammonium nitrate levels, and thus we cannot rule out compounding impacts or positive feedback loops between soil nutrients, fungi and ECM communities. *Melaleuca* cover was the only habitat variable strongly associated with the ECM community on French Island, with no associations on Phillip Island. The absence of strong trends across a range of genera was unexpected and in contrast to our hypotheses given the stark matrix of grassland (AM dominated) and woodland (ECM dominated) across both sites. Relict spores within soil (Carini et al. 2016; Okada and Matsuda 2022) may confound interpretations of the ‘active’ fungal community and requires further surveys to determine how vegetation may be influencing ECM communities.

### Aim 3: Comparison of fungal generalists on Phillip Island

Contrary to our predictions we detected only one ECM taxa in a single swamp wallaby scat from Phillip Island. Previous studies have demonstrated that swamp wallabies consume both epigeous and truffle-like fungi (Claridge et al. 2001; Vernes and McGrath 2009; Danks 2011; O’Malley 2012; Nest et al. 2023), and that fungi form a component of a ‘typical’ swamp wallaby diet across habitat types and seasons (Claridge et al. 2001; Vernes 2010). Critically, wallabies have also been found to consume truffle-like fungi at a comparable diversity to that of fungal specialists (Nuske et al. 2017). Previous studies recorded peaks in fungal consumption during winter (Vernes 2010; Danks 2011) and associations with short-term rainfall (Nest et al. 2023). Although higher than average rainfall was recorded during our trapping period (Australian Bureau of Meteorology 2025), we cannot rule out a seasonal bias, or the role of alternative food resources. Given Phillip Island is dominated by a matrix of grassland (AM dominated) and *Melaleuca* woodland (ECM dominated), we would expect both fluctuations in ECM fungal availability and abundance of alternative food resources (wallabies are known to browse and graze vegetation, *sensu* Fischer 2018).

Although bandicoot scats from Phillip Island had a lower ECM richness than those from French Island, the limited ECM detections in wallaby scats suggests that bandicoots may have the capacity to fulfill a vacant spore dispersal niche. *Russula* was a dominant component of the fungal community in bandicoot scats and contains both truffle-like and epigeous fruiting habits (Bougher and Lebel 2001; Nuske et al. 2018). Although, taxonomic resolution did not enable us to identify truffle-like taxa in *Russula* OTUs, bandicoots on Phillip Island may be performing a substantial role aiding in truffle-like *Russula* spore dispersal. Our results suggest that habitat characteristics are likely to be influencing patterns in fungal diet. Areas with lower graminoid cover, higher woody debris and herb cover, and higher percent cover of *Acacia* and *Gaudium* were associated with higher ECM richness in scats. As ECM fungi are obligate plant symbionts (Tedersoo et al. 2010), the limited ECM plant diversity across the Summerland Peninsula, and large areas of grassland are likely to reduce ECM fungal availability and bandicoot reliance on fungi as a food resource.

### Aim 4: Comparison of a fungal generalist and specialist on French Island

In contrast to Nuske et al. (2017) review of mycophagy and our hypothesis, we recorded no difference in ECM richness between bandicoot and potoroo scats, although their community composition differed. This may be explained by the small number of potoroo scat samples collected that limited our capacity to fully characterise their fungal diet (common for DNA metabarcoding studies, Nuske et al. 2018). As such, we note that, particularly for potoroo, additional scat sampling is required to sufficiently capture their fungal diet and compare this to co-occurring bandicoots. However, our results align with previous studies which showed potoroos to be highly mycophagous and we anticipate that the breadth of ECM fungi in the potoroo diet to increase with increased sampling (Bennett and Baxter 1989; Claridge et al. 1992, 1993a, b; Tory et al. 1997). ECM and truffle-like taxa were detected in a higher proportion of potoroo than bandicoot samples, and we recorded higher relative abundance of truffle-like fungi in potoroo scats. This suggests potoroos are likely to consume a greater abundance of fungi than bandicoots, although their potential for ECM spore dispersal was similar in our study. The high dominance of ECM in potoroo diet may also reflect a positive feedback loop where they enhance fungal dispersal, increasing availability and facilitating increased foraging resources for the mycophagous community (Schickmann et al. 2012).

Differences between bandicoots and potoroos may be driven by habitat preferences with bandicoots showing a tendency to forage in open vegetation, and potoroos in more closed vegetation (Eketone et al. 2026). Alternatively, bandicoot and potoroo may be foraging in comparable habitat yet selecting for different fungal species based on dietary preferences (Elliott et al. 2022b), or the availability and/or preference for alternative food resources. Fungal specialists, such as the potoroo, are expected to exhibit more consistent fungal consumption, and dependence on fungi across individuals and habitats (Bennet and Baxter 1989). The absence of clear trends between ECM richness or community composition with habitat attributes may suggest that both species are moving between ECM and non-ECM dominated habitats to forage. These movement patterns emphasise their potential role in dispersing ECM fungal spores across habitats (*sensu* Naccarella et al. 2026). The transport of ECM fungal spores from ECM to AM dominated areas can facilitate establishment of ECM plants (Elliott et al. 2022b), which can be critical in successional landscapes (Cázares and Trappe 1994; Terwilliger and Pastor 1999; Ashkannejhad and Horton 2006; Vernes and Trappe 2007; Vernes and Dunn 2009). Furthermore, mycopahgous mammals may play beneficial roles in restoration areas (Frank et al. 2006), post-disturbance landscapes (e.g., post-fire [Bennett and Baxter 1989; Johnson 1995 ] or post-timber harvesting [Stephens et al. 2021 ], by reintroducing ECM fungi [Aguirre et al. 2021 ]). The degree to which dispersers may shift fungal-plant communities is dependent on the diversity of fungi they consume (Stephens and Rowe 2020), foraging distances and habitat preferences of the animal disperser (Stephens et al. 2021). In Central Europe, small mammals with broader ecological niches were more influential in the dispersal of ECM inoculum beyond forested areas (Komur et al. 2021). Similarly, mammals that can persist in both undisturbed and disturbed sites (i.e., logging, agriculture, habitat fragmentation) had the capacity to facilitate the establishment and regeneration of native pine stands in west-central Mexico (Gil-Fernández et al. 2025). In this sense, fungal generalists, such as the bandicoot, by way of displaying fewer habitat and dietary preferences, and frequently moving between habitats, may play keystone roles for fungal dispersal across habitats and disturbance gradients.

#### Comparison of mycophagous communities

The absence of a full assemblage of mycophagous mammals (e.g., bandicoots, potoroos, rodents and dasyruids) across Phillip Island has likely resulted in a reduction in community-level dispersal functions, with likely negative impacts on the ECM soil community. In contrast, the mycophagous mammal community on French Island is relatively intact, including bush rat and swamp rat, both of which are native fungal generalists. Generalist rodents have been found to be equivalent to specialist species, in relation to the diversity and abundance of spores, when in high abundances in North America (Stephens and Rowe 2020). Given some rodent species can persist in disturbed areas their contribution could be substantial and underappreciated (Elliott et al. 2022a). Communities consisting of a range of dietary generalists tend to be more resilient to disturbances or long-term environmental change (Davey et al. 2012; Treloar et al. 2021; Kanishka et al. 2025). Hence, high functional redundancy, between generalists or generalist-specialists, may provide a buffer against extinctions or population fluctuations (Tylianakis et al. 2010). Phillip Island lacks a true fungal specialist with instead only fungal generalists such as the common brushtail possum (Nuske et al. 2019; Elliott et al. 2025; Kanishka et al. 2025) and invasive black rats (Vernes and McGrath 2009). Given long-nosed potoroos previously occurred on Phillip Island, last recorded in the 1980s, it is possible that key functional roles for ECM fungal dispersal have been lost (Phillip Island Nature Parks 2021). The residency time of spores within the soil however is unknown (Carini et al. 2016; Okada and Matsuda 2022), as such, possible legacy effects from long-nosed potoroo spore dispersal may have unknown effects on fungal communities on Phillip Island. Determining the mechanistic drivers of complementarity or redundancy across different ecosystems and mammal communities would enable a better understanding of the stability and resilience of dispersal networks in light of intensifying environmental changes (Schleuning et al. 2015; Dehling et al. 2021).

### Trends in non-native fungi

Biological invasions create novel ecological interactions that can be either detrimental or beneficial. We recorded non-native fungi, *Rhizopogon* and *Tuber*, in bandicoot and potoroo diet, reinforcing the flexible and opportunistic foraging patterns of native Australian mycopahges (Nuske et al. 2019; Naccarella et al. 2026). *Rhizopogon* and *Tuber* are northern hemisphere genera known to associate with *Pinaceae* (pine trees) and other exotic trees (Bonito et al. 2013). On French Island, we did not detect *Rhizopogon* or *Tuber* in soil samples, but pines are known to grow in the vicinity of the trapping site (~ 200 m) within the nightly foraging range of bandicoots (Rendall et al. 2018) and potoroos (Farmer et al. 2025). Thus, it is likely that bandicoot and potoroos are transporting those exotic spores around the island. The detection of non-native fungi highlights the potential for negative effects of fungal spore dispersal by facilitating the expansion of invasive fungi and plant partners. Our study adds to previous research on dispersal of non-native fungi by non-native mammals (Nuñez et al. 2013; Wood et al. 2015) and highlights that translocations may have both positive and negative impacts on species interactions and ecosystems, warranting post-translocation monitoring (e.g., Verdon et al. 2016; Coggan and Gibb 2019).

## Conclusion

Facilitating resilient species interaction networks and reinstating ecosystem function are considered key pathways and priorities for ecosystem restoration (Tylianakis et al. 2010; Byers 2022). Translocations offer a proactive management tool to restore lost ecosystem functions and mitigate the simplification of communities (Watson and Watson 2015; Seddon 2022; Evans et al. 2023). By comparing bandicoot scat with other co-occurring mycopahgous mammals, it appears bandicoots on Phillip and French Island may have the capacity to perform unique ECM spore dispersal roles, highlighting opportunities for synergistic benefits for the conservation of this species and ecosystem function across both sites. The detection of a single ECM taxa in wallaby scats from Phillip Island reinforces the variability in fungal generalist diets across species and between populations of the same species. We caution that single time point studies, such as this, may bias interpretations of the degree of mycophagy, particularly in generalist species and in areas where large fluctuations in alternative food resources are likely to shift dependency on fungal resources. Potoroo and bandicoot on French Island may perform complementary ECM dispersal roles, underscoring the importance of diverse mycophagous mammal communities in promoting diverse fungal communities (Vernes et al. 2015; Stephens et al. 2021; Nest et al. 2023).

We encourage the use of network analysis to better understand relationships between mycophagous mammals and fungi (*sensu* Vašutová et al. 2019). Integrating this understanding within a functional ecological view will enable greater understanding of the value of functionally diverse dispersal communities (Morán-López et al. 2020). In the context of translocation programs, we encourage comparative assessments of fungal diets across co-occurring species to identify the degree of complementarity or redundancy between endemic and translocated species. We further highlight the need to assess dispersal capacity at a community-scale across a range of site contexts to understand intraspecific variability, and how these patterns may differ across disturbance gradients. Translocations have the capacity to re-establish fungal spore dispersal networks among other ecosystem functions, directly contributing to restoration efforts. Quantifying the efficacy of translocations to restoration of ecosystem function and reinstatement of key species interactions requires an ecosystem-level, cross-taxa, and network-based approach.

## Supplementary Information

Below is the link to the electronic supplementary material.

Supplementary Material 1

## Data Availability

The data is available at https://doi.org/10.5281/zenodo.17823821
