# Supplementary material for: One species does not replace a community: functional roles of co-occurring translocated and endemic mycophagous mammals
Source: Oecologia. 2026 Aug 10;208(9):112. doi: 10.1007/s00442-026-05947-w (PMC13457359; doi:10.1007/s00442-026-05947-w)
Supplement: Supplementary file 1 [file 442_2026_5947_MOESM1_ESM.docx]

***One species does not replace a community: dispersal functions of translocated and relict mycophagous mammals***

**Supplementary Material**

## **Appendix S1**

Trap site locations were previously established as part of annual bandicoot population monitoring which has occurred at Summerland Peninsula, Phillip Island and Bluegums, French Island since the bandicoots were translocated. Traps were arranged in a series of grids (hereafter “blocks”) consisting of either 9 or 12 traps ~70 m apart (Block size: ~200 x 150 m on Phillip Island and ~100 x 150m on French island) (Figure 1). Both sites were trapped for three consecutive nights, with a total of 198 trap nights on Phillip Island and 288 trap nights on French Island.

Within each block of traps 20 x 20 m quadrats measured coarse scale vegetation variables (% cover of woody vegetation (trees and shrubs) and canopy cover). These quadrats were arranged in an "X" pattern across each grid of traps, where quadrats were placed at each of the four grid corners and at the centre-point (Phillip Island n = 30, French Island n =40) (Figure 1). Quadrats were oriented north-south, with a minimum of 20 m between quadrats and 10 m from any tracks or infrastructure. In each 20 m quadrat we also measured the basal diameter (at ground level) of all dominant canopy trees and shrubs capable of forming ECM associations. To measure fine scale variables (percent cover of graminoid, herb, moss, bare ground, woody debris, and litter) we then nested five 1 x 1 m quadrats within each 20m x 20m quadrat, one at each corner and one in the centre (Figure 1). The 1 m quadrats were grouped by each 20 m quadrat by taking the mean across the five quadrats for each variable and then grouped per block by taking the mean across 20 m quadrats for analysis purposes. For analysis, ECM plants were grouped by genus by taking the mean basal diameter within each quadrat and block.

**Figure S1.1.** (a)Location of eastern barred bandicoot (*Perameles gunnii*) translocation study sites, (b) example of soil sampling and vegetation survey design, and arrangement of trapping grids at (c) Phillip Island and (d) French Island.

## **Appendix S2**

In relation to *Aim 1*, observed ECM and truffle-like richness models were run with Generalised Linear Mixed Models (GLMM) with a negative binomial distribution (due to an overdispersed Poisson distribution; *lme4*, Bates et al 2015) and ECM Shannon diversity with a Linear Mixed Models (LMM) (Bates *et al.* 2015). We did not run a truffle-like Shannon diversity model due to low detections of truffle-like taxa. We were not able to compare truffle-like community composition between soil and bandicoot samples from Phillip Island, nor between bandicoot samples between Phillip and French Island due to low detections of truffle-like fungi in bandicoot samples from Phillip Island (*n* = 1).

In relation to *Aim 2*, the soil properties and vegetation composition models were run with a GLMM with a negative binomial distribution. Both ECM plant cover models and the French Island ECM plant basal diameter model were run with a GLMM with a negative binomial distribution We identified several outliers within the Phillip Island ECM plant cover model. We re-ran models without outliers and detected no change in the effects. Thus, we report the model with outliers retained. For the French Island ECM plant cover and ECM plant basal diameter models one outlier was identified per model and both were removed prior to analysis. The Phillip Island ECM plant basal diameter model was run with a Generalised Additive Model (GAM) as we identified non-linear patterns in residuals (Wood 2011). We applied a thin plate spline smoothers to both variables, *Allocasuarina* and *Melaleuca*.

In relation to Aim 3, the ECM richness models were run with GLMMs with a Poisson distribution. For the *bioenv* and db-RDA models, due to low sample sizes, only three ECM plant cover variables met our criteria, two of which were correlated. Therefore, we elected to run separate models with each combination of variables. We were not able to run *bioenv* or db-RDA models for ECM plant basal diameter model as only two plant genera were present in more than two blocks and were correlated with one another.

In relation to Aim 4, observed ECM richness was modelled with a GLMM with a negative binomial distribution, and ECM Shannon diversity and truffle-like richness with LMMs. For all vegetation models a GLMM with a negative binomial distribution was used. We identified several outliers within the vegetation composition and ECM plant cover models. We re-ran models without outliers and detected no change in the direction of the result. Thus, we report models with outliers retained. For the ECM plant basal diameter model, we identified three outliers. We re-ran models and detected a change in the direction of the results. Thus, we report models with outliers removed.

## **Appendix S3**

Rarefaction OTU accumulation curves indicated that for a given number of reads soil samples contained more ECM and truffle-like OTUs per read count than bandicoot samples on Phillip Island (Appendix 1; Figure S1.5). On French Island, bandicoot samples contained more ECM OTUs per read count than soil samples followed by potoroo samples (Appendix 1; Figure S1.6). For truffle-like ECM, soil samples similarly contained more OTUs per read count than soil and potoroo samples, however soil and potoroo samples contained comparable truffle-like OTUs (Appendix 1; Figure S1.6). MultSE estimates showed that our results for bandicoot samples from Phillip Island and potoroo samples from French Island should be interpreted with caution, as neither were approaching an asymptote based on our current sample sizes and samples contained high variability suggesting that our sampling was not adequate to reflect dietary breadth.

**Figure S3.1.** Multivariate pseudo standard error (as per Anderson and Santana‐Garcon 2015) estimated for incremental number of samples in soil, eastern barred bandicoot (*Perameles gunnii)* scat and swamp wallaby (*Wallabia bicolor*) scat collected from Phillip Island for (a) all operational taxonomic units and (b) ectomycorrhizal (ECM) operational taxonomic units. A single ECM OTU was present in swamp wallaby scat samples.

**Figure S3.2.** Multivariate pseudo standard error (as per Anderson and Santana‐Garcon 2015) estimated for incremental number of samples in soil, eastern barred bandicoot (*Perameles gunnii)* scat*,* and long-nosed potoroo (*Potorous tridactylus trisulcatus*) scat collected from French Island for (a) all operational taxonomic units and (b) ectomycorrhizal operational taxonomic units.

**Figure S3.3.** Rarefied accumulation curves ± SE for (a) ectomycorrhizal (ECM) and (b) truffle-like operational taxonomic units from soil (*n*= 29), eastern barred bandicoot (*Perameles gunnii*) (*n* = 8) scat or swamp wallaby (*Wallabia bicolor*) (*n*=20) scat samples collected from Phillip Island.

**Figure S3.4.** Rarefied accumulation curves ± SE for (a) ectomycorrhizal (ECM) and (b) truffle-like operational taxonomic units from soil (*n* = 38), eastern barred bandicoot (*Perameles gunnii*) (*n* = 37) and long-nose potoroo (*Potorous tridactylus trisulcatus*) (*n*=6) scat collected from French Island.

**Figure S3.5.** Location of soil, eastern barred bandicoot (*Perameles gunnii*) and swamp wallaby scat (*Wallabia bicolor*) collected from Phillip Island. Colours indicate the observed ectomycorrhizal (ECM) richness of each sample. Soil *n* = 28, bandicoot scat *n* = 8, wallaby *n* = 20. Map excludes samples with low read count <500.

**Figure S3.6.** Location of samples collected of soil, eastern barred bandicoot (*Perameles gunnii*) and long-nosed potoroo scat (*Potorous tridactylus trisulcatus*) from French Island. Colours indicate the observed ectomycorrhizal (ECM) richness of each sample. Soil *n* = 38 and bandicoot scat *n* = 37, potoroo *n* = 6. Map excludes samples with low read count <500.

**Figure S3.7.** Relative abundance (µ ± SE) of operational taxonomic units in soil, eastern barred bandicoot (*Perameles gunnii*) scat, long nosed potoroo (*Potorous tridactylus trisulcatus*) scat and swamp wallaby (*Wallabia bicolor*) scat samples collected from Phillip (a) and French (b) Islands in Victoria, southeastern Australia.

**Figure S3.8.** Ectomycorrhizal (a) and truffle-like (b) observed richness and Shannon diversity of soil, eastern barred bandicoot (*Perameles gunnii*) scat, long nosed potoroo (*Potorous tridactylus trisulcatus*) scat and swamp wallaby (*Wallabia bicolor*) scat collected across Phillip and French Islands in Victoria, southeastern Australia. Black circles indicate outliers. Total sequenced sample size: Phillip Island: bandicoot scat *n* = 8, wallaby *n* = 20, soil *n* = 29. French Island: bandicoot scat *n* = 37, potoroo scat *n* = 6, soil *n* = 38 soil. Shannon diversity plots exclude samples with no detection of ectomycorrhizal or truffle-like fungi.

**Figure S3.9.** Relative abundance of ectomycorrhizal genera in soil, eastern barred bandicoot (*Perameles gunnii*) scat, long nosed potoroo (*Potorous tridactylus trisulcatus*) scat and swamp wallaby (*Wallabia bicolor*) scat collected from Phillip and French Island, Victoria, southeastern Australia. Columns represent each individual sample which contains ectomycorrhizal genera. Total sequenced sample size: Phillip Island: bandicoot scat *n* = 8, wallaby *n* = 20, soil *n* = 29. French Island: bandicoot scat *n* = 37, potoroo scat *n* = 6, soil *n* = 38 soil. Asterix indicates genera with a truffle-like (hypogeous) fruiting habit. Plus indicates genera that have both an epigeous and truffle-like fruiting habit. Tringle indicates genera that are not-native to Australia.

## **Appendix 4 - Aim 1**

**Table S4.1.** Summary of Tukey post-hoc tests based on predicted response variables from comparing observed richness and shannon diversity of ectomycorrhizal (ECM) between Phillip and French Island, and between sample types (soil and eastern barred bandicoot (EBB, *Perameles gunnii)*). Numbers in bold indicate significant (p <0.05) variables. Observed ECM richness was modelled with a Generalised Linear Mixed Effects Models (GLMMs) and ECM shannon diversity with a linear mixed model (LMM).

|  | Site | Sample Type | Est. | SE | | z-ratio | p-value | Marginal R^2^ | Conditional R^2^ |
| --- | --- | --- | --- | --- | --- | --- | --- | --- | --- |
| Observed ECM Richness | Phillip Island | Soil - EBB | -2.400 | 0.710 | | -3.387 | **<0.001** | 0.238 | 0.244 |
|  | French Island | Soil - EBB | 1.350 | 0.386 | | 3.509 | **<0.001** |  |  |
|  | Soil | French Island – Phillip Island | -1.850 | 0.391 | | -4.722 | **<0.001** |  |  |
|  | EBB | French Island – Phillip Island | 1.910 | 0.692 | | 2.763 | **0.006** |  |  |
|  | **Site** | **Sample Type** | **Est.** | **SE** | **df** | **t- ratio** | **p-value** | **Marginal R^2^** | **Conditional R^2^** |
| ECM Shannon Diversity | Phillip Island | Soil - EBB | -1.201 | 0.436 | 67.300 | -2.753 | **0.008** | 0.160 | 0.160 |
|  | French Island | Soil - EBB | 0.741 | 0.307 | 70.000 | 2.418 | **0.018** |  |  |
|  | Soil | French Island – Phillip Island | -0.568 | 0.302 | 26.800 | -1.880 | 0.071 |  |  |
|  | EBB | French Island – Phillip Island | 1.375 | 0.442 | 49.100 | 3.108 | **0.003** |  |  |

**Table S4.2.** Summary of Tukey post-hoc tests based on predicted response variables from a Generalised Linear Mixed Effects Models (GLMMs) comparing observed richness of truffle-like ectomycorrhizae between Phillip and French Island and sample types (soil and eastern barred bandicoot (EBB, *Perameles gunnii*)). Numbers in bold indicate significant (p <0.05) variables.

|  | Site | Sample Type | Est. | SE | z-ratio | p-value | Marginal R^2^ | Conditional R^2^ |
| --- | --- | --- | --- | --- | --- | --- | --- | --- |
| Observed truffle-like Richness | Phillip Island | Soil - EBB | -0.956 | 0.842 | -1.134 | 0.257 | 0.275 | 0.275 |
|  | French Island | Soil - EBB | 0.622 | 0.324 | 1.919 | 0.055 |  |  |
|  | Soil | French Island – Phillip Island | -1.120 | 0.320 | -3.494 | **<0.001** |  |  |
|  | EBB | French Island – Phillip Island | 0.460 | 0.844 | 0.545 | 0.586 |  |  |

**Table S4.3.** Summary of Tukey post-hoc tests based on predicted response variables from Generalised Linear Mixed Effects Models or a Linear Mixed Model (ECM Shannon diversity model) comparing observed richness or shannon diversity of ectomycorrhizal (ECM) and truffle-like ECM between Phillip and French Island and sample types (soil and eastern barred bandicoot (EBB, *Perameles gunnii*)).

|  | Response | Site | Sample Type | EMM | SE | df | lower.CL | upper.CL |
| --- | --- | --- | --- | --- | --- | --- | --- | --- |
| ECM | Observed Richness | Phillip Island | Soil | 2.758 | 0.312 | NA | 2.146 | 3.370 |
|  |  |  | EBB | 0.355 | 0.644 | NA | -0.908 | 1.620 |
|  |  | French Island | Soil | 0.913 | 0.302 | NA | 0.321 | 1.510 |
|  |  |  | EBB | 2.268 | 0.255 | NA | 1.768 | 2.770 |
|  | Shannon Diversity | Phillip Island | Soil | 1.433 | 0.189 | 16.800 | 1.034 | 1.830 |
|  |  |  | EBB | 0.232 | 0.397 | 62.800 | -0.562 | 1.020 |
|  |  | French Island | Soil | 0.865 | 0.236 | 37.500 | 0.388 | 1.340 |
|  |  |  | EBB | 1.606 | 0.192 | 14.100 | 1.188 | 2.020 |
| Truffle-like | Observed Richness | Phillip Island | Soil | 1.649 | 0.158 | NA | 1.338 | 1.960 |
|  |  |  | EBB | 0.693 | 0.827 | NA | -0.928 | 2.310 |
|  |  | French Island | Soil | 0.531 | 0.278 | NA | -0.014 | 1.080 |
|  |  |  | EBB | 1.153 | 0.167 | NA | 0.826 | 1.480 |

**Table S4.4.** Summary of pairwise comparisons from Permutational Multivariate Analysis of Variance (PERMANOVA) and Analysis of Variance (ANOVA) on dispersion test (PERMDISP) comparing ectomycorrhizal (ECM) or truffle-like ECM community composition between sample type (soil and eastern barred bandicoot (*Perameles gunnii*) scat) and site (French and Phillip Island), using Raup-Crick dissimilarity. Numbers in bold indicate significant (p<0.05) variables.

|  |  |  | **df** | **SS** | **MS** | **Pseudo-F** | **p-value** |
| --- | --- | --- | --- | --- | --- | --- | --- |
| PERMANOVA | ECM | Sample Type | 1, 60 | 0.539 | 0.539 | 2.049 | 0.144 |
|  |  | Site | 1, 60 | 0.973 | 0.973 | 3.239 | 0.050 |
|  |  | Sample Type*Site | 1, 60 | 0.989 | 0.989 | 3.762 | **0.013** |
|  | Truffle-like | Sample Type | 1, 27 | 0.588 | 0.588 | 3.246 | 0.060 |
|  |  | Site | 1, 27 | 1.032 | 1.032 | 5.254 | **0.006** |
|  |  | Sample Type*Site | 1, 27 | 0.377 | 0.377 | 2.082 | 0.164 |
|  |  |  | **t-value** | | **p-value** | | |
| Dispersion test | ECM | Sample Type | 3.265 | | **0.002** | | |
|  |  | Site | 2.467 | | **0.018** | | |
|  | Truffle-like | Sample Type | 2.743 | | **0.014** | | |
|  |  | Site | 1.229 | | 0.265 | | |

**Table S4.5.** Summary of Permutational Multivariate Analysis of Variance (PERMANOVA) and Analysis of Variance (ANOVA) on dispersion test (PERMDISP) comparing ectomycorrhizal (ECM) or truffle-like ECM community composition in soil samples between Phillip and French Island, using Raup-Crick dissimilarity of presence/absence data. Numbers in bold indicate significant (p <0.05) variables.

| **Sample Type** | **Sites** |  |  | **df** | **SS** | **F** | **R^2^** | **Pr(>F)** |
| --- | --- | --- | --- | --- | --- | --- | --- | --- |
| Soil | Phillip Island – French Island | PERMANOVA | ECM | 1, 41 | 1.091 | 0.076 | 3.390 | **0.022** |
|  |  |  | Truffle-like | 1, 22 | 1.537 | 6.596 | 0.231 | **0.005** |
|  |  | Dispersion test | ECM | 1, 30 | 0.011 | 0.011 | 0.378 | 0.542 |
|  |  |  | Truffle-like | 1, 22 | 0.086 | 0.086 | 0.507 | 0.484 |

**Table S4.6.** Summary of Permutational Multivariate Analysis of Variance (PERMANOVA) and Analysis of Variance (ANOVA) on dispersion test (PERMDISP) comparing ectomycorrhizal community composition in eastern barred bandicoot (*Perameles gunnii*) scat between Phillip and French Island, and between soil and eastern barred bandicoot (*Perameles gunnii*) scat on Phillip Island, using Raup-Crick dissimilarity of presence/absence data. Numbers in bold indicate significant (p <0.05) variables.

| **Sample Type** | **Sites** |  | **t-value** | **p-value** |
| --- | --- | --- | --- | --- |
| Eastern barred bandicoot | Phillip Island – French Island | PERMANOVA | 1.747 | 0.105 |
|  |  | Dispersion test | 0.485 | 0.690 |
| Soil - EBB | Phillip Island | PERMANOVA | 1.765 | **0.033** |
|  |  | Dispersion test | 1.613 | 0.233 |

**Table S4.7.** Summary of Permutational Multivariate Analysis of Variance (PERMANOVA) and Analysis of Variance (ANOVA) on dispersion test (PERMDISP) comparing ectomycorrhizal community composition between soil and eastern barred bandicoot (*Perameles gunnii*) scat on French Island, using Raup-Crick dissimilarity of presence/absence data. Numbers in bold indicate significant (p <0.05) variables.

| **Site** | **Sample Type** |  |  | **df** | **SS** | **MS** | **Pseudo-F** | **p-value** |
| --- | --- | --- | --- | --- | --- | --- | --- | --- |
| French Island | Soil - EBB | PERMANOVA | ECM | 1,35 | 0.518 | 0.518 | 2.129 | 0.146 |
|  |  |  | Truffle-like | 1,19 | 0.323 | 0.323 | 2.180 | 0.140 |
|  |  |  |  | **df** | **F** | | **p-value** | |
|  |  | Dispersion test | ECM | 1,42 | 2.717 | | 0.104 | |
|  |  |  | Truffle-like | 1,26 | 0.683 | | 0.479 | |

**Figure S4.1.** Non-metric multidimensional scaling (NMDS) ordination of (a) ectomycorrhizal and (b) truffle-like fungal communities (using Raup-Crick dissimilarities based on presence/absence) in soil and eastern barred bandicoot (*Perameles gunnii*) scat collected from French and Phillip Island. Truffle-like fungi were only detected in a single bandicoot scat from Phillip Island; thus, we present truffle-like communities in soil only. Dashed lines represent 95% confidence interval.

**Table S4.8.** Ectomycorrhizal and truffle-like genera that are associated with sample type (soil and eastern barred bandicoot (*Perameles gunnii*) scat) and site (Phillip and French Island) using Indicator Species Analysis. IV represents the indicator species value. IV-A (component A) represents the probability that the surveyed site belongs to the target site group given the fact that the genus has been found (i.e., specificity). IV-B (Component B) represents the probability of finding the genus in sites belonging to the specified site groups (i.e., fidelity).

|  | **Group** | **Genera** | **Comp A** | **Comp B** | **Stat** | **p-value** |
| --- | --- | --- | --- | --- | --- | --- |
| ALL Ectomycorrhizal | French Island - Eastern Barred bandicoot | *Rhizopogon* | 1.000 | 0.222 | 0.471 | 0.040 |
|  | Phillip Island – Soil | *Pisolithus* | 1.000 | 0.308 | 0.555 | 0.013 |
|  |  | *Cenococcum* | 1.000 | 0.308 | 0.555 | 0.013 |
|  |  | *Sphaerosoma* | 1.000 | 0.269 | 0.519 | 0.010 |
|  |  | *Tuber* | 0.838 | 0.308 | 0.508 | 0.047 |
|  | Phillip Island – Eastern Barred bandicoot | *Russula* | 0.939 | 0.833 | 0.885 | <0.001 |
|  | French Island - Eastern Barred bandicoot & Phillip Island – Soil | *Ruhlandiella* | 0.958 | 0.434 | 0.645 | 0.027 |
|  |  | *Thelephora* | 1.000 | 0.283 | 0.532 | 0.048 |
|  | French Island - Eastern Barred bandicoot, French Island – Soil & Phillip Island – Soil | *Tomentella* | 1.000 | 0.686 | 0.828 | 0.036 |
| Truffle-like | French Island – Eastern barred bandicoot, Phillip Island - Eastern barred bandicoot & Phillip Island - Soil | *Ruhlandiella* | 0.969 | 0.706 | 0.827 | 0.015 |

## **Appendix 5 – Aim 2**

**Figure S5.1.** Non-metric multidimensional scaling (nMDS) ordination of ECM plant percent cover (based on 20 m^2^ quadrats) on Phillip and French Islands. Dashed lines represent 95% confidence interval. Community composition is significantly different between sites (F = 6.184, R^2^ = 0.114, p <0.001).

**Table S5.1.** Models describing the relationship between observed ectomycorrhizal richness in soil in relation to site (Phillip and French Island) and soil nutrients. Models shown include those with ΔAICc <2. Reported are the number of parameters within the model (K), degrees of freedom (df), log likelihood, Akaike’s information criteria corrected for small sample sizes (AICc), the difference from the model with lowest AICc (ΔAICc) and model AICc weights (AICc ω).

| **Model** | **K** | **df** | **Log likelihood** | **AICc** | **ΔAICc** | **AICc** ω |
| --- | --- | --- | --- | --- | --- | --- |
| Site + Ammonium nitrate + Nitrate nitrogen + Site *Nitrate nitrogen | 4 | 6 | -161.186 | 335.8 | 0 | 0.110 |
| Site + Ammonium nitrate + Nitrate nitrogen + Site *Nitrate nitrogen + Organic carbon | 5 | 7 | -160.693 | 337.3 | 1.52 | 0.051 |
| Site + Ammonium nitrate + Nitrate nitrogen + Site *Nitrate nitrogen + Site * Ammonium nitrate | 5 | 7 | -160.821 | 337.6 | 1.78 | 0.045 |

**Table S5.2**. Generalized Linear Models (GLM) describing observed ectomycorrhizal richness in soil in relation to site (Phillip and French Island) and soil nutrients. Numbers in bold indicate significant (p <0.05) variables.

| **Model** | **Variables** | **Estimate** | **Std. Error** | **z value** | **Pr(>\|z\|)** |
| --- | --- | --- | --- | --- | --- |
| Site + Ammonium nitrate + Nitrate nitrogen + Site *Nitrate nitrogen | Intercept | -0.178 | 0.395 | -0.450 | 0.653 |
|  | Site | 2.713 | 0.455 | 5.965 | **<0.001** |
|  | Ammonium nitrate | -0.834 | 0.207 | -4.039 | **<0.001** |
|  | Nitrate nitrogen | -2.219 | 0.726 | -3.058 | **0.002** |
|  | Site(Phillip Island)*Nitrate nitrogen | 2.017 | 0.826 | 2.442 | **0.015** |
| Site + Ammonium nitrate + Nitrate nitrogen + Site *Nitrate nitrogen + Organic carbon | Intercept | -0.022 | 0.391 | -0.057 | 0.955 |
|  | Site | 2.362 | 0.502 | 4.704 | **<0.001** |
|  | Ammonium nitrate | -0.919 | 0.219 | -4.206 | **<0.001** |
|  | Nitrate nitrogen | -2.109 | 0.684 | -3.082 | **0.002** |
|  | Organic Carbon | 0.232 | 0.213 | 1.091 | 0.275 |
|  | Site(Phillip Island)*Nitrate nitrogen | 1.793 | 0.797 | 2.250 | **0.024** |
| Site + Ammonium nitrate + Nitrate nitrogen + Site*Nitrate nitrogen + Site * Ammonium nitrate | Intercept | -0.181 | 0.385 | -0.471 | 0.637 |
|  | Site | 2.697 | 0.445 | 6.056 | **<0.001** |
|  | Ammonium nitrate | -1.008 | 0.298 | -3.385 | **0.001** |
|  | Nitrate nitrogen | -2.180 | 0.701 | -3.111 | **0.002** |
|  | Site(Phillip Island)* Nitrate nitrogen | 1.712 | 0.835 | 2.051 | **0.040** |
|  | Site(Phillip Island)* Ammonium nitrate | 0.384 | 0.416 | 0.921 | 0.357 |

**Figure S5.2.** Relationship between (a) Ammonium nitrate and observed ectomycorrhizal richness within soil from Phillip and French Island, and (b) Nitrate nitrogen and observed ectomycorrhizal richness within soil from Phillip and French Island.

**Table S5.3.** Soil properties identified in each rank-order correlation (via *bioenv*) with the ectomycorrhizal community within soil from Phillip Island. Sulphur and organic carbon were removed due to high correlation with other soil properties.

| **Variables** | **size** | **correlation** |
| --- | --- | --- |
| Ammonium nitrate | 1 | 0.153 |
| Ammonium nitrate, Nitrate nitrogen | 2 | 0.186 |
| Ammonium nitrate, Nitrate nitrogen, Phosphorus | 3 | 0.217 |
| Ammonium nitrate, Nitrate nitrogen, Phosphorus, Potassium | 4 | 0.225 |
| Ammonium nitrate, Nitrate nitrogen, Phosphorus, Potassium, Conductivity | 5 | 0.171 |
| Ammonium nitrate, Nitrate nitrogen, Phosphorus, Potassium, Conductivity, pH | 6 | 0.128 |

**Table S5.4.** Summary of coefficient associated with each explanatory variable on each axis determined by a distance-based Redundancy Analysis (db-RDA), using a Raup-Crick dissimilarity of presence/absence data of ectomycorrhizal community in soil samples and soil nutrient variables from Phillip Island.

| **Variables** | **dbRDA1** | **dbRDA2** | **dbRDA3** | **dbRDA4** |
| --- | --- | --- | --- | --- |
| Ammonium nitrate | -0.050 | 0.673 | -0.596 | 0.435 |
| Nitrate nitrogen | 0.347 | 0.026 | -0.563 | 0.749 |
| Phosphorus | 0.905 | 0.333 | -0.092 | -0.250 |
| Potassium | 0.519 | 0.718 | 0.245 | 0.393 |

**Table S5.5.** Summary of Analysis of Variance of ectomycorrhizal community in soil and soil nutrients from Phillip Island via distance-based Redundancy Analysis, using a Raup-Crick dissimilarity of presence/absence data. Numbers in bold indicate significant (p0.05) variables.

| **Variables** | **df** | **SS** | **F** | **p-value** |
| --- | --- | --- | --- | --- |
| Ammonium nitrate | 1 | 0.420 | 1.458 | 0.254 |
| Nitrate nitrogen | 1 | 0.505 | 1.752 | 0.174 |
| Phosphorus | 1 | 0.894 | 3.103 | **0.011** |
| Potassium | 1 | 0.312 | 1.083 | 0.360 |

**Table S5.6.** Soil properties identified in each rank-order correlation (via *bioenv*) with the ectomycorrhizal community within soil from French Island. Conductivity was removed due to high correlation with other soil properties.

| **Variables** | **size** | **correlation** |
| --- | --- | --- |
| pH | 1 | 0.251 |
| pH, Phosphorus | 2 | 0.313 |
| pH, Phosphorus, Organic carbon | 3 | 0.375 |
| pH, Phosphorus, Organic carbon, Sulphur | 4 | 0.365 |
| pH, Phosphorus, Organic carbon, Sulphur, Ammonium nitrate | 5 | 0.333 |
| pH, Phosphorus, Organic carbon, Sulphur, Ammonium nitrate, Nitrate nitrogen | 6 | 0.241 |
| pH, Phosphorus, Organic carbon, Sulphur, Ammonium nitrate, Nitrate nitrogen, Potassium | 7 | 0.197 |

**Table S5.7.** Summary of coefficient associated with each explanatory variable on each axis determined by a distance-based Redundancy Analysis (db-RDA), using a Raup-Crick dissimilarity of presence/absence data of ectomycorrhizal community in soil and soil nutrient from French Island.

| **Variables** | **dbRDA1** | **dbRDA2** | **dbRDA3** |
| --- | --- | --- | --- |
| Phosphorus | 0.417 | 0.199 | -0.887 |
| Organic Carbon | -0.008 | 1.000 | 0.015 |
| pH | -0.838 | -0.344 | -0.423 |

**Table S5.8.** Summary of Analysis of Variance of ectomycorrhizal community in soil and soil properties from French Island via a distance-based Redundancy Analysis using a Raup-Crick dissimilarity of presence/absence data. Numbers in bold indicate significant (p <0.05) variables.

| **Variables** | **df** | **SS** | **F** | **p** |
| --- | --- | --- | --- | --- |
| Phosphorus | 1 | 0.389 | 1.281 | 0.333 |
| Organic Carbon | 1 | 0.568 | 1.872 | 0.123 |
| pH | 1 | 0.729 | 2.402 | 0.067 |

**Figure S5.3.** Distance-based Redundancy Analyses (db-RDA) of ectomycorrhizal community in soil samples collected from Phillip and French Island, Victoria, southeastern Australia. Vector overlays show soil properties with the maximum (rank) correlations as calculated via bioenv and models for each site separately. Phillip Island: Ammonium nitrate, nitrate nitrogen, phosphorous, and potassium, bioenv correlation = 0.225. French Island: Phosphorous, organic carbon and pH, bioenv correlation = 0.375.

**Table S5.9.** Models describing the relationship between observed ectomycorrhizal richness in soil samples in relation to site (Phillip and French Island) and vegetation composition variables (% cover). Models shown include those with ΔAICc <2. Reported are the number of parameters within the model (K), degrees of freedom (df), log likelihood, Akaike’s information criteria corrected for small sample sizes (AICc), the difference from the model with lowest AICc (ΔAICc) and model AICc weights (AICc ω). Litter was deemed an uninformative parameter as per Leroux (2019).

| **Model** | **K** | **df** | **Log likelihood** | **AICc** | **ΔAICc** | **AICc** ω |
| --- | --- | --- | --- | --- | --- | --- |
| Bare ground + Site + Woody Vegetation + Herb*Site + Herb | 5 | 7 | -165.761 | 347.4 | 0 | 0.048 |
| Bare ground + Site + Woody Vegetation + Herb*Site + Herb + Woody Vegetation*Site | 6 | 8 | -164.686 | 347.9 | 0.43 | 0.038 |
| Bare ground + Site + Woody Vegetation + Herb*Site + Herb + Bareground*Site | 6 | 8 | -165.006 | 348.5 | 1.07 | 0.028 |
| Bare ground + Site + Woody Vegetation + Herb*Site + Herb + Litter | 6 | 8 | -165.008 | 348.5 | 1.08 | 0.028 |
| Bare ground + Site + Woody Vegetation + Herb*Site + Woody Vegetation*Site | 5 | 6 | -167.741 | 348.9 | 1.46 | 0.023 |
| Bare ground + Site + Woody Vegetation + Herb*Site | 4 | 5 | -168.951 | 348.9 | 1.47 | 0.023 |

**Table S5.10.** Generalized Linear Models (GLM) describing observed ectomycorrhizal richness in soil in relation to site (Phillip and French Island) and coarse vegetation variables (% cover). Numbers in bold indicate significant (p<0.05) variables.

| **Model** | **Variables** | **Estimate** | **Std. Error** | **z value** | **Pr(>\|z\|)** |
| --- | --- | --- | --- | --- | --- |
| Bare ground + Site + Woody Vegetation + Herb*Site + Herb | Intercept | -0.274 | 0.298 | -0.921 | 0.357 |
|  | Site (Phillip Island) | 3.230 | 0.414 | 7.796 | **<0.001** |
|  | Woody vegetation | 0.563 | 0.224 | 2.519 | **0.012** |
|  | Herb | 0.398 | 0.263 | 1.512 | 0.130 |
|  | Bare ground | 0.648 | 0.211 | 3.078 | **0.002** |
|  | Phillip Island*Herb | -1.077 | 0.386 | -2.791 | **0.005** |
| Bare ground + Site + Woody Vegetation + Herb*Site + Herb + Woody Vegetation*Site | Intercept | -0.425 | 0.331 | -1.285 | 0.199 |
|  | Site (Phillip Island) | 3.187 | 0.426 | 7.481 | **<0.001** |
|  | Woody vegetation | 0.753 | 0.279 | 2.703 | **0.007** |
|  | Herb | 0.440 | 0.266 | 1.655 | 0.098 |
|  | Bare ground | 0.645 | 0.208 | 3.108 | **0.002** |
|  | Phillip Island *Herb | -1.029 | 0.390 | -2.637 | **0.008** |
|  | Phillip Island * Woody vegetation | -0.649 | 0.383 | -1.692 | 0.091 |
| Bare ground + Site + Woody Vegetation + Herb*Site + Herb + Bareground*Site | Intercept | -0.386 | 0.321 | -1.204 | 0.229 |
|  | Site (Phillip Island) | 3.164 | 0.425 | 7.450 | **<0.001** |
|  | Woody vegetation | 0.604 | 0.234 | 2.581 | **0.010** |
|  | Herb | 0.438 | 0.264 | 1.662 | 0.097 |
|  | Bare ground | 0.751 | 0.214 | 3.503 | **<0.001** |
|  | Phillip Island *Herb | -1.129 | 0.386 | -2.927 | **0.003** |
|  | Phillip Island *Bare ground | -0.599 | 0.504 | -1.189 | 0.235 |
| Bare ground + Site + Woody Vegetation + Herb*Site + Herb + Litter | Intercept | -0.399 | 0.308 | -1.296 | 0.195 |
|  | Site (Phillip Island) | 3.416 | 0.430 | 7.936 | **<0.001** |
|  | Woody vegetation | 0.721 | 0.235 | 3.075 | **0.002** |
|  | Herb | 0.478 | 0.261 | 1.828 | 0.068 |
|  | Bare ground | 0.671 | 0.209 | 3.214 | **0.001** |
|  | Litter | -0.262 | 0.173 | -1.509 | 0.131 |
|  | Phillip Island *Herb | -1.233 | 0.393 | -3.135 | **0.002** |
| Bare ground + Site + Woody Vegetation + Herb*Site + Woody Vegetation*Site | Intercept | -0.425 | 0.331 | -1.285 | 0.199 |
|  | Site (Phillip Island) | 3.187 | 0.426 | 7.481 | **<0.001** |
|  | Woody vegetation | 0.753 | 0.279 | 2.703 | **0.007** |
|  | Bare ground | 0.645 | 0.208 | 3.108 | **0.002** |
|  | French Island*Herb | 0.440 | 0.266 | 1.655 | 0.098 |
|  | Phillip Island *Herb | -0.589 | 0.286 | -2.059 | **0.040** |
|  | Phillip Island *Woody vegetation | -0.649 | 0.383 | -1.692 | 0.091 |
| Bare ground + Site + Woody Vegetation + Herb*Site | Intercept | -0.274 | 0.298 | -0.921 | 0.357 |
|  | Site (Phillip Island) | 3.230 | 0.414 | 7.796 | **<0.001** |
|  | Woody vegetation | 0.563 | 0.224 | 2.519 | **0.012** |
|  | Bare ground | 0.648 | 0.211 | 3.078 | **0.002** |
|  | French Island*Herb | 0.398 | 0.263 | 1.512 | 0.130 |
|  | Phillip Island *Herb | -0.679 | 0.280 | -2.425 | **0.015** |

**Figure S5.4.** Relationship between observed ectomycorrhizal richness within soil from Phillip and French Island and percent cover of (a) woody vegetation, (b) bare ground, and (c) herb in relation to site.

**Table S5.11.** Models describing the relationship between observed ectomycorrhizal richness in soil samples in relation to ECM plant cover variables (% cover per genera) on Phillip Island. Models shown include those with ΔAICc <2. Reported are the number of parameters within the model (K), degrees of freedom (df), log likelihood, Akaike’s information criteria corrected for small sample sizes (AICc), the difference from the model with lowest AICc (ΔAICc) and model AICc weights (AICc ω).

| **Model** | **K** | **df** | **Log likelihood** | **AICc** | **ΔAICc** | **AICc** ω |
| --- | --- | --- | --- | --- | --- | --- |
| *Melaleuca* | 1 | 3 | -106.090 | 219.1 | 0 | 0.187 |
| Null Model | 0 | 2 | -107.591 | 219.6 | 0.50 | 0.146 |
| *Acacia* | 1 | 3 | -106.398 | 219.8 | 0.61 | 0.138 |
| *Melaleuca, Acacia* | 2 | 4 | -105.086 | 219.8 | 0.70 | 0.132 |

**Table S5.12.** Generalized Linear Models describing observed ectomycorrhizal richness in soil in relation to ECM plant cover variables (% cover per genera) on Phillip Island. Numbers in bold indicate significant (p <0.05) variables.

| **Model** | **Variables** | **Estimate** | **Std. Error** | **z value** | **Pr(>\|z\|)** |
| --- | --- | --- | --- | --- | --- |
| *Melaleuca* | Intercept | 2.688 | 0.242 | 11.090 | **<0.001** |
|  | *Melaleuca* | 0.445 | 0.244 | 1.820 | 0.069 |

**Table S5.13.** Models describing the relationship between observed ectomycorrhizal richness in soil in relation to ECM plant basal diameter (per genera) on Phillip Island. Models shown include those with ΔAICc <2. Reported are the number of parameters within the model (K), degrees of freedom (df), log likelihood, Akaike’s information criteria corrected for small sample sizes (AICc), the difference from the model with lowest AICc (ΔAICc) and model AICc weights (AICc ω). Gaudium was removed due to high correlation with other plant genera.

| **Model** | **K** | **df** | **Log likelihood** | **AICc** | **ΔAICc** | **AICc** ω |
| --- | --- | --- | --- | --- | --- | --- |
| *Allocasuarina* + *Melaleuca* | 2 | 5 | -120.855 | 256.7 | 0 | 0.417 |
| *Melaleuca* | 1 | 3 | -123.785 | 256.8 | 0.1 | 0.397 |

**Table S5.14.** General Additive Model (GAM) describing observed ectomycorrhizal richness in soil samples in relation to ECM plant basal diameter (per genera) on Phillip Island. Numbers in bold indicate significant (p <0.05) variables. Thin plate spline smoothers were applied to *Allocasuarina* and *Melaleuca* variables.

|  | **Variables** | **Estimate** | **Std. Error** | **t value** | **Pr(>\|z\|)** |
| --- | --- | --- | --- | --- | --- |
| *Allocasuarina* + *Melaleuca* | Intercept | 16.103 | 3.172 | 5.076 | **<0.001** |
|  |  | **edf** | **Ref.df** | **F** | **p-value** |
|  | *Allocasuarina* | 1.834 | 1.972 | 2.208 | 0.137 |
|  | *Melaleuca* | 1.926 | 1.994 | 4.784 | **0.017** |
| *Melaleuca* | Intercept | 16.103 | 3.179 | 4.765 | **<0.001** |
|  |  | **edf** | **Ref.df** | **F** | **p-value** |
|  | *Melaleuca* | 1.856 | 1.979 | 3.141 | 0.066 |

**Figure S5.5.** Relationship between observed ectomycorrhizal richness within soil samples from Phillip basal diameter of *Melaleuca* (p = 0.017).

**Table S5.15.** Vegetation variables identified in each rank-order correlation (via *bioenv*) with the ectomycorrhizal community within soil samples and vegetation variables from Phillip Island. Woody debris, litter and canopy were removed due to high correlation with other vegetation composition variables.

|  | **Variables** | **size** | **correlation** |
| --- | --- | --- | --- |
| Vegetation composition (% cover) | Graminoid | 1 | 0.048 |
|  | Graminoid, Herb | 2 | 0.072 |
|  | Graminoid, Herb, Bareground | 3 | 0.048 |
|  | Graminoid, Herb, Bareground, Woody vegetation | 4 | 0.025 |
| ECM plant cover variables (% cover per genera) | *Allocasuarina* | 1 | 0.062 |
|  | *Allocasuarina, Gaudium* | 2 | 0.123 |
|  | *Allocasuarina, Gaudium, Acacia* | 3 | 0.131 |
|  | *Allocasuarina, Gaudium, Acacia, Melaleuca* | 4 | 0.074 |
| ECM plant basal diameter (per genera) | *Melaleuca* | 1 | 0.065 |
|  | *Melaleuca, Allocasuarina* | 2 | 0.105 |
|  | *Melaleuca, Allocasuarina, Gaudium* | 3 | 0.114 |
|  | *Melaleuca, Allocasuarina, Gaudium, Acacia* | 4 | 0.111 |

**Table S5.16.** Models describing the relationship between observed ectomycorrhizal richness in soil in relation to ECM plant cover (% cover per genera) on French Island. Models shown include those with ΔAICc <2. Reported are the number of parameters within the model (K), degrees of freedom (df), log likelihood, Akaike’s information criteria corrected for small sample sizes (AICc), the difference from the model with lowest AICc (ΔAICc) and model AICc weights (AICc ω).

| **Model** | **K** | **df** | **Log likelihood** | **AICc** | **ΔAICc** | **AICc** ω |
| --- | --- | --- | --- | --- | --- | --- |
| *Eucalyptus* + *Melaleuca* | 2 | 4 | -57.282 | 123.8 | 0 | 0.326 |
| *Eucalyptus* + *Melaleuca* + *Leptospermum* | 3 | 5 | -56.416 | 124.8 | 0.95 | 0.202 |
| *Eucalyptus* + *Melaleuca* + *Gaudium* | 3 | 5 | -56.513 | 125.0 | 1.15 | 0.184 |

**Table S5.17.** Generalized Linear Models describing observed ectomycorrhizal richness in soil in relation to ECM plant cover (% cover per genera) on French Island. Numbers in bold indicate significant (p <0.05) variables.

| **Model** | **Variables** | **Estimate** | **Std. Error** | **z value** | **Pr(>\|z\|)** |
| --- | --- | --- | --- | --- | --- |
| *Eucalyptus* + *Melaleuca* | Intercept | 0.089 | 0.248 | 0.358 | 0.720 |
|  | *Eucalyptus* | 0.670 | 0.175 | 3.828 | **<0.001** |
|  | *Melaleuca* | 1.191 | 0.189 | 6.288 | **<0.001** |
| *Eucalyptus* + *Melaleuca* + *Leptospermum* | Intercept | 0.045 | 0.249 | 0.182 | 0.855 |
|  | *Eucalyptus* | 0.624 | 0.170 | 3.678 | **<0.001** |
|  | *Melaleuca* | 1.276 | 0.196 | 6.511 | **<0.001** |
|  | *Leptospermum* | 0.336 | 0.226 | 1.489 | 0.137 |
| *Eucalyptus* + *Melaleuca* + *Gaudium* | Intercept | 0.050 | 0.251 | 0.197 | 0.843 |
|  | *Eucalyptus* | 0.697 | 0.173 | 4.029 | **<0.001** |
|  | *Melaleuca* | 1.241 | 0.190 | 6.541 | **<0.001** |
|  | *Gaudium* | 0.241 | 0.197 | 1.225 | 0.220 |

**Figure S5.6.** Relationship between observed ectomycorrhizal richness within soil from French Island and percent cover of (a) *Eucalyptus*, and (b) *Melaleuca*.

**Table S5.18.** Models describing the relationship between observed ectomycorrhizal richness in soil in relation to ECM plant basal diameter (per genera) on French Island. Models shown include those with ΔAICc <2. Reported are the number of parameters within the model (K), degrees of freedom (df), log likelihood, Akaike’s information criteria corrected for small sample sizes (AICc), the difference from the model with lowest AICc (ΔAICc) and model AICc weights (AICc ω).

| **Model** | **K** | **df** | **Log likelihood** | **AICc** | **ΔAICc** | **AICc** ω |
| --- | --- | --- | --- | --- | --- | --- |
| *Eucalyptus* + *Leptospermum* + *Melaleuca* | 3 | 5 | -59.568 | 131.1 | 0 | 0.588 |
| *Eucalyptus* + *Melaleuca* | 2 | 4 | -61.346 | 131.9 | 0.87 | 0.381 |

**Table S5.19.** Generalized Linear Mixed Effects Models describing observed ectomycorrhizal richness in soil in relation to ECM plant basal diameter (per genera) on French Island. Numbers in bold indicate significant (p <0.05) variables.

| **Model** | **Variables** | **Estimate** | **Std. Error** | **z value** | **Pr(>\|z\|)** |
| --- | --- | --- | --- | --- | --- |
| *Eucalyptus* + *Leptospermum* + *Melaleuca* | Intercept | 0.182 | 0.254 | 0.717 | 0.474 |
|  | *Melaleuca* | 0.985 | 0.213 | 4.617 | **<0.001** |
|  | *Leptospermum* | 0.371 | 0.205 | 1.813 | 0.070 |
|  | *Eucalyptus* | 0.671 | 0.195 | 3.448 | **0.001** |
| *Eucalyptus* + *Melaleuca* | Intercept | 0.282 | 0.276 | 1.024 | 0.306 |
|  | *Eucalyptus* | 1.085 | 0.244 | 4.456 | **<0.001** |
|  | *Melaleuca* | 0.645 | 0.230 | 2.812 | **0.005** |

**Figure S5.7.** Relationship between observed ectomycorrhizal richness within soil from French Island and basal diameter of (a) *Eucalyptus*, and (b) *Melaleuca*.

**Table S5.20.** Soil nutrient variables identified in each rank-order correlation (via *bioenv*) with the ectomycorrhizal community within soil and vegetation composition (% cover) variables from French Island. Graminoid and litter were removed due to high correlation with other vegetation composition variables.

| **Variables** | **size** | **correlation** |
| --- | --- | --- |
| Herb | 1 | 0.251 |
| Herb, Woody vegetation | 2 | 0.253 |
| Herb, Woody vegetation, Canopy | 3 | 0.258 |
| Herb, Woody vegetation, Canopy, Woody debris | 4 | 0.274 |
| Herb, Woody vegetation, Canopy, Woody debris, Bareground | 5 | 0.198 |

**Table S5.21.** Summary of coefficient associated with each explanatory variable on each axis determined by a distance-based Redundancy Analysis (db-RDA), using a Raup-Crick dissimilarity of presence/absence data of ectomycorrhizal community in soil samples and vegetation composition (% cover) variables from French Island.

| **Variables** | **dbRDA1** | **dbRDA2** | **dbRDA3** | **dbRDA4** |
| --- | --- | --- | --- | --- |
| Woody vegetation | 0.725 | 0.480 | -0.038 | 0.492 |
| Herb | -0.905 | 0.324 | 0.105 | 0.254 |
| Woody debris | 0.412 | 0.833 | 0.347 | -0.127 |
| Canopy | 0.584 | 0.376 | -0.719 | -0.033 |

**Table S5.22.** Analysis of Variance of ectomycorrhizal community in soil and vegetation composition (% cover) from French Island via a distance-based Redundancy Analysis, using a Raup-Crick dissimilarity of presence/absence data. Numbers in bold indicate significant (p <0.05) variables.

| **Variables** | **df** | **SS** | **F** | **p** |
| --- | --- | --- | --- | --- |
| Woody vegetation | 1 | 0.596 | 1.726 | 0.158 |
| Herb | 1 | 0.560 | 1.620 | 0.210 |
| Woody debris | 1 | 0.168 | 0.485 | 0.775 |
| Canopy | 1 | 0.163 | 0.470 | 0.789 |

**Table S5.23.** ECM plant genera identified in each rank-order correlation (via *bioenv*) with the ectomycorrhizal community within soil and ECM plant cover (% cover per genera) variables from French Island.

| **Variables** | **size** | **correlation** |
| --- | --- | --- |
| *Eucalyptus* | 1 | 0.114 |
| *Eucalyptus, Melaleuca* | 2 | 0.135 |
| *Eucalyptus, Melaleuca, Leptospermum* | 3 | 0.213 |
| *Eucalyptus, Melaleuca, Leptospermum, Allocasuarina* | 4 | 0.207 |
| *Eucalyptus, Melaleuca, Leptospermum, Allocasuarina, Acacia* | 5 | 0.163 |
| *Eucalyptus, Melaleuca, Leptospermum, Allocasuarina, Acacia, Gaudium* | 6 | 0.091 |

**Table S5.24.** Summary of coefficient associated with each explanatory variable on each axis determined by a distance-based Redundancy Analysis (dbRDA), using a Raup-Crick dissimilarity of presence/absence data of ectomycorrhizal community in soil and ECM plant cover (% cover per genera) from French Island.

| **Variables** | **dbRDA1** | **dbRDA2** | **dbRDA3** |
| --- | --- | --- | --- |
| *Melaleuca* | 0.993 | 0.007 | -0.121 |
| *Leptospermum* | -0.602 | 0.044 | -0.797 |
| *Eucalyptus* | -0.273 | -0.961 | 0.054 |

**Table S5.25.** Analysis of Variance of ectomycorrhizal community in soil and ECM plant cover (% cover per genera) from French Island via a distance-based Redundancy Analysis using a Raup-Crick dissimilarity of presence/absence data. Numbers in bold indicate significant (p <0.05) variables.

| **Variables** | **df** | **SS** | **F** | **p** |
| --- | --- | --- | --- | --- |
| *Melaleuca* | 1 | 1.120 | 3.745 | **0.012** |
| *Leptospermum* | 1 | 0.214 | 0.716 | 0.625 |
| *Eucalyptus* | 1 | 0.412 | 1.377 | 0.248 |

**Table S5.26.** ECM plant genera identified in each rank-order correlation (via *bioenv*) with the ectomycorrhizal community within soil and ECM plant basal diameter (per genera) variables from French Island.

| **Variables** | **size** | **correlation** |
| --- | --- | --- |
| *Eucalyptus* | 1 | 0.114 |
| *Eucalyptus, Melaleuca* | 2 | 0.105 |
| *Eucalyptus, Melaleuca, Gaudium* | 3 | 0.116 |
| *Eucalyptus, Melaleuca, Gaudium, Acacia* | 4 | 0.084 |
| *Eucalyptus, Melaleuca, Gaudium, Acacia, Allocasuarina* | 5 | 0.033 |
| *Eucalyptus, Melaleuca, Gaudium, Acacia, Allocasuarina, Leptospermum* | 6 | -0.102 |

## **Appendix 6 – Aim 3**

**Table S6.1.** Summary of vegetation composition (% cover) variables in relation to eastern barred bandicoot scat on Phillip Island, and the results of a Principal Components Analysis (PCA) identifying the variation accounted for by each component, including loadings, eigenvalue and variance explained. Blank values indicate components >0 or <0.1.

| **Variable** | **PC1** | **PC2** | **PC3** | **PC4** | **PC5** | **PC6** | **PC7** |
| --- | --- | --- | --- | --- | --- | --- | --- |
| BG | 0.454 | 0.269 | 0.113 |  | 0.437 |  | 0.719 |
| WD | 0.141 | -0.649 | 0.259 | 0.380 | -0.430 | 0.153 | 0.375 |
| LITTER | 0.409 | 0.260 | -0.453 | -0.223 | -0.680 | -0.178 | 0.129 |
| HERB | -0.172 | -0.423 | -0.766 | 0.110 | 0.309 | -0.239 | 0.200 |
| GRAMINOID | -0.346 | 0.497 |  | 0.756 | -0.157 |  | 0.141 |
| WOODY | 0.483 | -0.109 | 0.168 | 0.321 | 0.139 | -0.681 | -0.375 |
| Canopy | 0.474 |  | -0.306 | 0.345 | 0.151 | 0.643 | -0.354 |
| **Eigenvalue** | 4.081 | 2.046 | 8.730e-01 | 3.622e-16 | 3.227e-16 | 5.321e-17 | 0 |
| **% variance explained** | 58.295 | 29.234 | 12.471 | 5.175e-17 | 4.611e-17 | 7.602e-18 | 0 |

**Table S6.2.** Generalized Linear Mixed Model describing observed ectomycorrhizal richness in eastern barred bandicoot scat in relation to vegetation composition (% cover) variables on Phillip Island. Numbers in bold indicate significant (p<0.05) variables.

| **Variables** | **Estimate** | **Std. Error** | **z value** | **Pr(>\|z\|)** |
| --- | --- | --- | --- | --- |
| Intercept | 0.199 | 0.357 | 0.557 | 0.577 |
| PC1 | 0.002 | 0.190 | 0.010 | 0.992 |
| PC2 | -0.344 | 0.164 | -2.095 | **0.036** |

**Figure S6.1.** Relationship between observed ectomycorrhizal richness within eastern barred bandicoot (*Perameles gunni*) samples from Phillip Island and PC2 representing a gradient of high percent cover of graminoid, and low percent cover of woody debris and herb.

**Table S6.3.** Generalized Linear Mixed Model describing observed ectomycorrhizal richness in eastern barred bandicoot (*Perameles gunni*) scat in relation to ECM plant cover (% cover per genera) on Phillip Island. Numbers in bold indicate significant (p<0.05) variables.

| **Model** | **Variables** | **Estimate** | **Std. Error** | **z value** | **Pr(>\|z\|)** |
| --- | --- | --- | --- | --- | --- |
| 1 | Intercept | 0.597 | 0.419 | 1.424 | 0.154 |
|  | *Melaleuca* | -0.016 | 0.021 | -0.762 | 0.446 |
| 2 | Intercept | -0.011 | 0.412 | -0.027 | 0.978 |
|  | *Acacia* | 0.225 | 0.105 | 2.147 | **0.032** |
| 3 | Intercept | -0.154 | 0.466 | -0.330 | 0.741 |
|  | *Gaudium* | 0.248 | 0.122 | 2.034 | **0.042** |

**Figure S6.2.** Relationship between observed ectomycorrhizal richness within eastern barred bandicoot (*Perameles gunnii*) samples from Phillip Island and percent cover of (a) *Acacia* and (b) *Gaudium*.

**Table S6.4.** Generalized Linear Mixed Models (GLMM) describing observed ectomycorrhizal richness in scat (bandicoot and potoroo) in relation to ECM plant basal diameter (per genera) on Phillip Island. Numbers in bold indicate significant (p<0.05) variables.

| **Model** | **Variables** | **Estimate** | **Std. Error** | **z value** | **Pr(>\|z\|)** |
| --- | --- | --- | --- | --- | --- |
| 1 | Intercept | 1.035 | 0.483 | 2.142 | **0.032** |
|  | *Melaleuca* | -0.008 | 0.006 | -1.537 | 0.124 |
| 3 | Intercept | 0.200 | 0.395 | 0.506 | 0.613 |
|  | *Gaudium* | 0.015 | 0.019 | 0.786 | 0.432 |

**Table S6.5.** Vegetation composition variables identified in each rank-order correlation (via *bioenv*) with the ectomycorrhizal community within soil and vegetation composition (% cover) from Phillip Island.

| **Variables** | **size** | **correlation** |
| --- | --- | --- |
| Woody debris | 1 | 0.451 |
| Woody debris, Woody vegetation | 2 | 0.451 |
| Woody debris, Woody vegetation, Herb | 3 | 0.118 |

**Table S6.6.** ECM plant genera identified in each rank-order correlation (via *bioenv*) with the ectomycorrhizal community within soil and ECM plant cover (% cover per genera) from Phillip Island.

|  | **Variables** | **size** | **correlation** |
| --- | --- | --- | --- |
| Model 1 | *Gaudium* | 1 | 0.501 |
|  | *Gaudium*, *Melaleuca* | 2 | 0.080 |
| Model 2 | *Acacia* | 1 | 0.415 |
|  | *Acacia*, *Melaleuca* | 2 | 0.328 |

## **Appendix 7 – Aim 4**

**Table S7.1.** Summary of Tukey post-hoc tests comparing observed richness and shannon diversity of ectomycorrhizal (ECM) between sample types from French Island. Numbers in bold indicate significant (p <0.05) variables. Observed ECM richness was modelled with a Generalised Linear Mixed Effects Models, and ECM shannon diversity with a linear mixed model (LMM).

|  | Site | Sample Type | Est. | SE | | z-ratio | p-value | Marginal R^2^ | Conditional R^2^ |
| --- | --- | --- | --- | --- | --- | --- | --- | --- | --- |
| Observed ECM Richness | French Island | Soil - EBB | 1.333 | 0.373 | | 3.569 | **0.001** | 0.148 | 0.148 |
|  |  | Soil - LNP | 1.241 | 0.697 | | 1.781 | 0.176 |  |  |
|  |  | EBB - LNP | 0.091 | 0.693 | | 0.131 | 0.991 |  |  |
|  | **Site** | **Sample Type** | **Est.** | **SE** | **df** | **t- ratio** | **p-value** | **Marginal R^2^** | **Conditional R^2^** |
| ECM Shannon Diversity | French Island | Soil - EBB | 0.741 | 0.281 | 45.900 | 2.641 | **0.030** | 0.141 | 0.141 |
|  |  | Soil - LNP | 0.226 | 0.423 | 46.900 | 0.535 | 0.855 |  |  |
|  |  | EBB - LNP | 0.515 | 0.399 | 46.400 | 1.289 | 0.408 |  |  |

**Table S7.2.** Summary of Tukey post-hoc tests based on predicted response variables from a linear mixed model comparing observed richness of truffle-like ectomycorrhizal (ECM) between sample type from French Island. Sample type was set as fixed effects and block was included as a random effect. Numbers in bold indicate significant (p <0.05) variables.

|  | Site | Sample Type | Est. | SE | t-ratio | p-value | Marginal R^2^ | Conditional R^2^ |
| --- | --- | --- | --- | --- | --- | --- | --- | --- |
| Observed truffle-like Richness | French Island | Soil - EBB | 0.917 | 1.060 | 28.600 | 0.862 | 0.124 | 0.124 |
|  |  | Soil - LNP | 1.467 | 0.785 | 28.000 | 1.869 |  |  |
|  |  | EBB - LNP | 0.550 | 1.180 | 27.500 | 0.467 |  |  |

**Table S7.3.** Summary of Tukey post-hoc tests based on predicted response variables from Generalised Linear Mixed Effects Models (GLMMs) or Linear Mixed Models (LMM) comparing observed richness or shannon diversity of ectomycorrhizal (ECM) and truffle-like ECM between sample types from French Island.

|  | Response | Site | Sample Type | EMM | SE | df | lower.CL | upper.CL |
| --- | --- | --- | --- | --- | --- | --- | --- | --- |
| ECM | Observed Richness | French Island | Soil | 0.937 | 0.258 | NA | 0.409 | 1.470 |
|  |  |  | EBB | 2.270 | 0.269 | NA | 1.763 | 2.780 |
|  |  |  | LNP | 2.170 | 0.643 | NA | 0.918 | 3.440 |
|  | Shannon Diversity | French Island | Soil | 1.606 | 0.181 | 10.700 | 1.207 | 2.010 |
|  |  |  | EBB | 1.092 | 0.367 | 41.900 | 0.351 | 1.830 |
|  |  |  | LNP | 0.865 | 0.214 | 26.700 | 0.426 | 1.300 |
| Truffle-like | Observed Richness | French Island | Soil | 1.700 | 0.611 | 18.100 | 0.417 | 2.980 |
|  |  |  | EBB | 3.170 | 0.453 | 11.300 | 2.172 | 4.160 |
|  |  |  | LNP | 2.250 | 0.975 | 25.500 | 0.245 | 4.250 |

**Table S7.4.** Summary of pairwise comparisons from Permutational Multivariate Analysis of Variance (PERMANOVA) and ANOVA on dispersion test (PERMDISP) comparing ectomycorrhizal community composition between sample types on French Island, using Raup-Crick dissimilarity of presence/absence data. Numbers in bold indicate significant (p <0.05) variables.

|  |  |  | **t-value** | **p-value** |
| --- | --- | --- | --- | --- |
| PERMANOVA | ECM | Soil - EBB | 1.459 | 0.144 |
|  |  | Soil - LNP | 1.779 | 0.052 |
|  |  | EBB - LNP | 2.500 | **0.003** |
|  | Truffle-like | Soil - EBB | 1.676 | 0.105 |
|  |  | Soil - LNP | 0.425 | 0.629 |
|  |  | EBB - LNP | 2.690 | **0.019** |
| Dispersion test | ECM | Soil - EBB | 1.648 | 0.113 |
|  |  | Soil - LNP | 2.021 | 0.078 |
|  |  | EBB - LNP | 1.411 | 0.224 |
|  | Truffle-like | Soil - EBB | 0.192 | 0.872 |
|  |  | Soil - LNP | 0.979 | 0.833 |
|  |  | EBB - LNP | 0.914 | 0.592 |

**Figure S7.1.** Non-metric multidimensional scaling (NMDS) ordination of (a) ectomycorrhizal fungal communities and (b) truffle-like communities (using Raup-Crick dissimilarities based on presence/absence) in soil, eastern barred bandicoot (*Perameles gunnii*) and long-nosed potoroo (*Potorous tridactylus trisulcatus*) collected from French Island. Dashed lines represent 95% confidence interval.

**Table S7.5.** Models describing the relationship between observed ectomycorrhizal richness in scat (eastern barred bandicoot and long-nosed potoroo) in relation to vegetation composition (% cover) on French Island. Models shown include those with ΔAICc <2. Reported are the number of parameters within the model (K), degrees of freedom (df), log likelihood, Akaike’s information criteria corrected for small sample sizes (AICc), the difference from the model with lowest AICc (ΔAICc) and model AICc weights (AICc ω).

| **Model** | **K** | **df** | **Log likelihood** | **AICc** | **ΔAICc** | **AICc** ω |
| --- | --- | --- | --- | --- | --- | --- |
| Null Model | 0 | 3 | -138.851 | 284.3 | 0 | 0.205 |
| Woody vegetation | 1 | 4 | -138.800 | 286.7 | 2.34 | 0.064 |

**Table S7.6.** Models describing the relationship between observed ectomycorrhizal richness in scat (eastern barred bandicoot and long-nosed potoroo) in relation to ECM plant cover (% cover per genera) on French Island. Models shown include those with ΔAICc <2. Reported are the number of parameters within the model (K), degrees of freedom (df), log likelihood, Akaike’s information criteria corrected for small sample sizes (AICc), the difference from the model with lowest AICc (ΔAICc) and model AICc weights (AICc ω).

| **Model** | **K** | **df** | **Log likelihood** | **AICc** | **ΔAICc** | **AICc** ω |
| --- | --- | --- | --- | --- | --- | --- |
| Null Model | 0 | 3 | -138.846 | 284.3 | 0 | 0.254 |
| *Leptospermum* | 1 | 4 | -138.724 | 286.5 | 2.19 | 0.085 |

**Table S7.7.** Models describing the relationship between observed ectomycorrhizal richness in scat (eastern barred bandicoot and long-nosed potoroo) in relation to ECM plant basal diameter (per genera) on French Island. Models shown include those with ΔAICc <2. Reported are the number of parameters within the model (K), degrees of freedom (df), log likelihood, Akaike’s information criteria corrected for small sample sizes (AICc), the difference from the model with lowest AICc (ΔAICc) and model AICc weights (AICc ω).

| **Model** | **K** | **df** | **Log likelihood** | **AICc** | **ΔAICc** | **AICc** ω |
| --- | --- | --- | --- | --- | --- | --- |
| Null Model | 0 | 3 | -128.526 | 263.7 | 0 | 0.246 |
| *Eucalyptus* | 1 | 4 | -128.344 | 265.8 | 2.11 | 0.085 |

**Table S7.8.** Vegetation variables identified in each rank-order correlation (via *bioenv*) model with the ectomycorrhizal community within scat (eastern barred bandicoot and long-nosed potoroo) from French Island. Graminoid and litter were removed due to high correlation with other vegetation composition variables.

|  | **Variables** | **size** | **correlation** |
| --- | --- | --- | --- |
| Vegetation composition (% cover) | Woody vegetation | 1 | -0.008 |
|  | Woody debris, Graminoid | 2 | 0.004 |
|  | Woody debris, Graminoid, Bareground | 3 | 0.007 |
|  | Woody debris, Graminoid, Bareground, Woody vegetation | 4 | 0.000 |
|  | Woody debris, Graminoid, Bareground, Woody vegetation, Herb | 5 | -0.015 |
| ECM plant cover (% cover per genera) | *Eucalyptus* | 1 | 0.052 |
|  | *Eucalyptus, Gaudium* | 2 | 0.075 |
|  | *Eucalyptus, Gaudium, Melaleuca* | 3 | 0.043 |
|  | *Eucalyptus, Gaudium, Melaleuca, Leptospermum* | 4 | 0.026 |
|  | *Eucalyptus, Gaudium, Melaleuca, Leptospermum, Acacia* | 5 | -0.049 |
| ECM plant basal diameter (per genera) | *Eucalyptus* | 1 | 0.052 |
|  | *Eucalyptus, Acacia* | 2 | 0.019 |
|  | *Eucalyptus, Acacia, Leptospermum* | 3 | 0.012 |
|  | *Eucalyptus, Acacia, Leptospermum, Melaleuca* | 4 | -0.053 |

**Table S7.9.** Ectomycorrhizal and truffle-like genera that are associated with samples using Indicator Species Analysis. IV represents the indicator species value. IV-A (component A) represents the probability that the surveyed site belongs to the target site group given the fact that the genus has been found (i.e., specificity). IV-B (Component B) represents the probability of finding the genus in sites belonging to the specified site groups (i.e., fidelity).

|  | **Group** | **Genera** | **Comp A** | **Comp B** | **Stat** | **p-value** |
| --- | --- | --- | --- | --- | --- | --- |
| ALL Ectomycorrhizal | Eastern Barred bandicoot | *Lactifluus* | 0.993 | 0.407 | 0.636 | 0.019 |
|  | Long-nosed potoroo | *Russula* | 0.892 | 0.833 | 0.862 | 0.007 |
|  |  | *Lactarius* | 0.937 | 0.667 | 0.790 | 0.000 |
|  |  | *Rhizopogon* | 0.993 | 0.333 | 0.575 | 0.028 |
|  |  | *Labyrinthomyces* | 0.922 | 0.333 | 0.554 | 0.028 |
|  |  | *Tylopilus* | 0.853 | 0.333 | 0.533 | 0.023 |
| Truffle-like | Long-nosed potoroo | *Labyrinthomyces* | 0.912 | 0.500 | 0.675 | 0.032 |
